# Generation of Periventricular Reactive Astrocytes Overexpressing Aquaporin 4 Is Stimulated by Mesenchymal Stem Cell Therapy

**DOI:** 10.3390/ijms24065640

**Published:** 2023-03-15

**Authors:** María García-Bonilla, Betsaida Ojeda-Pérez, Kirill Shumilov, Luis-Manuel Rodríguez-Pérez, Dolores Domínguez-Pinos, Javier Vitorica, Sebastián Jiménez, Reposo Ramírez-Lorca, Miriam Echevarría, Casimiro Cárdenas-García, Teresa Iglesias, Antonia Gutiérrez, James P. McAllister, David D. Limbrick, Patricia Páez-González, Antonio J. Jiménez

**Affiliations:** 1 Department of Cell Biology, Genetics and Physiology, University of Malaga, 29010 Malaga, Spain; 2 Instituto de Investigación Biomédica de Málaga (IBIMA), 29010 Malaga, Spain; 3 Department of Pediatrics, Washington University in St. Louis School of Medicine, St. Louis, MO 63110, USA; 4 Departamento de Fisiología Humana, Histología Humana, Anatomía Patológica y Educación Física y Deportiva, University of Malaga, 29010 Malaga, Spain; 5 Department of Molecular Biology and Biochemistry, University of Seville, 41013 Sevilla, Spain; 6 Institute of Biomedicine of Seville (IBiS), Virgen del Rocío University Hospital, (HUVR)/Spanish National Research Council (CSIC)/University of Seville, 41013 Seville, Spain; 7 Centro de Investigación Biomédica en Red de Enfermedades Neurodegenerativas (CIBERNED), Instituto de Salud Carlos II, 28029 Madrid, Spain; 8 Department of Physiology and Biophysics, University of Seville, 41009 Seville, Spain; 9 Servicios Centrales de Apoyo a la Investigación (SCAI), University of Malaga, 29010 Malaga, Spain; 10 Instituto de Investigaciones Biomédicas “Alberto Sols”, Consejo Superior de Investigaciones Científicas-Universidad Autónoma de Madrid (CSIC-UAM), 28029 Madrid, Spain; 11 Department of Neurosurgery, Washington University in St. Louis School of Medicine, St. Louis, MO 63110, USA

**Keywords:** hydrocephalus, aquaporin 4, astrocyte reaction, mesenchymal stem cell, stem cell therapy, proteomic

## Abstract

Aquaporin-4 (AQP4) plays a crucial role in brain water circulation and is considered a therapeutic target in hydrocephalus. Congenital hydrocephalus is associated with a reaction of astrocytes in the periventricular white matter both in experimental models and human cases. A previous report showed that bone marrow-derived mesenchymal stem cells (BM-MSCs) transplanted into the lateral ventricles of hyh mice exhibiting severe congenital hydrocephalus are attracted by the periventricular astrocyte reaction, and the cerebral tissue displays recovery. The present investigation aimed to test the effect of BM-MSC treatment on astrocyte reaction formation. BM-MSCs were injected into the lateral ventricles of four-day-old hyh mice, and the periventricular reaction was detected two weeks later. A protein expression analysis of the cerebral tissue differentiated the BM-MSC-treated mice from the controls and revealed effects on neural development. In in vivo and in vitro experiments, BM-MSCs stimulated the generation of periventricular reactive astrocytes overexpressing AQP4 and its regulatory protein kinase D-interacting substrate of 220 kDa (Kidins220). In the cerebral tissue, mRNA overexpression of nerve growth factor (NGF), vascular endothelial growth factor (VEGF), hypoxia-inducible factor-1 (HIF1α), and transforming growth factor beta 1 (TGFβ1) could be related to the regulation of the astrocyte reaction and AQP4 expression. In conclusion, BM-MSC treatment in hydrocephalus can stimulate a key developmental process such as the periventricular astrocyte reaction, where AQP4 overexpression could be implicated in tissue recovery.

## 1. Introduction

Hydrocephalus, the most common disease treated by pediatric neurosurgeons, is a physiological disorder of the cerebrospinal fluid (CSF) typically associated with high intraventricular pressure (ICP) and a consequent expansion of the cerebral ventricles [1]. Pediatric hydrocephalus can be acquired or congenital, and in the latter case, obstruction of the cerebral aqueduct is one of the most common causes [1,2,3]. The pathophysiology of congenital obstructive hydrocephalus comprises alterations in the normal structure, physiology, and biochemistry of the brain, including ischemia/hypoxia, damage to cerebral white matter, inflammation, and microglial/astroglial reactions [4,5,6].

Current treatments for hydrocephalus are surgical and are restricted to alleviating ventricular pressure by drainage of CSF through ventricular shunts, third ventriculostomy, or extraventricular drains [7]. Because these treatments are only palliative and prevent some of the associated damage, new approaches or tools are needed to provide neuroprotection and promote the recovery or regeneration of damaged tissues [8]. The water channel aquaporin-4 (AQP4) is a crucial molecule in brain water transport. AQP4 is present in astrocytes and, thus, has been proposed as a therapeutic target in hydrocephalus [9,10,11,12,13,14].

Various stem cell therapies have been assayed in experimental forms of congenital and acquired feto-neonatal hydrocephalus. Neural stem cells [15] and mesenchymal stem cells [16,17] have shown promising results in hydrocephalus of obstructive and posthemorrhagic origins. In neurodegenerative diseases, bone marrow-derived mesenchymal stem cells (BM-MSCs) were shown to play a neuroprotective role due to their capacity to migrate to degenerated regions and produce different growth factors [18,19]. BM-MSCs can provide a neuroprotective effect by reducing oxidative stress, cell differentiation, and apoptosis, by increasing angiogenesis, or by modulating the inflammatory response [20,21].

The hyh mouse (hydrocephalus with hop gait) [22] has been used as a model to study obstructive congenital hydrocephalus etiopathogenesis. The hyh mouse has a mutation in the *Napa* gene, which affects the normal development of the neuroepithelium covering the brain ventricles. Therefore, an obstruction of the cerebral aqueduct induces severe hydrocephalus within the first days of postnatal development [23,24]. Then, several neuropathological events are triggered, such as myelin degeneration, glial reactions, a neuroexcitotoxic environment, and edema [25,26].

In a previous study in hyh mice, BM-MSCs were injected into the lateral ventricle, and they were attracted and hosted by reactive astrocytes of the white matter [17]. The periventricular astrocyte reaction is a key event in hydrocephalus development [6,25,27,28,29]. In humans and experimental animal models of hydrocephalus, it has been proposed that these astrocytes can form a barrier that replaces the absent ependyma and contribute to alleviating edema in the white matter through AQP4 expression [10,11,27,30]. Thus, the present investigation aimed to evaluate the effects of BM-MSC treatment on astrocyte reaction formation and AQP4 expression. For this purpose, BM-MSCs were injected into the lateral ventricle of four-day-old hyh mice before development of the astrocyte reaction [25] and were maintained for fourteen days post-treatment. BM-MSCs were detected to be integrated, surviving, and undifferentiated into reactive astrocytes in the periventricular white matter. BM-MSC transplantation stimulated the periventricular astrocyte reaction and thus overexpression of AQP4 in the white matter. Stimulation of the astrocyte reaction by BM-MSCs was also proven in vitro.

## 2. Results

### 2.1. BM-MSCs Injected into the Ventricles Integrate and Survive Fourteen Days in the Damaged Periventricular Walls

**Figure 1:**
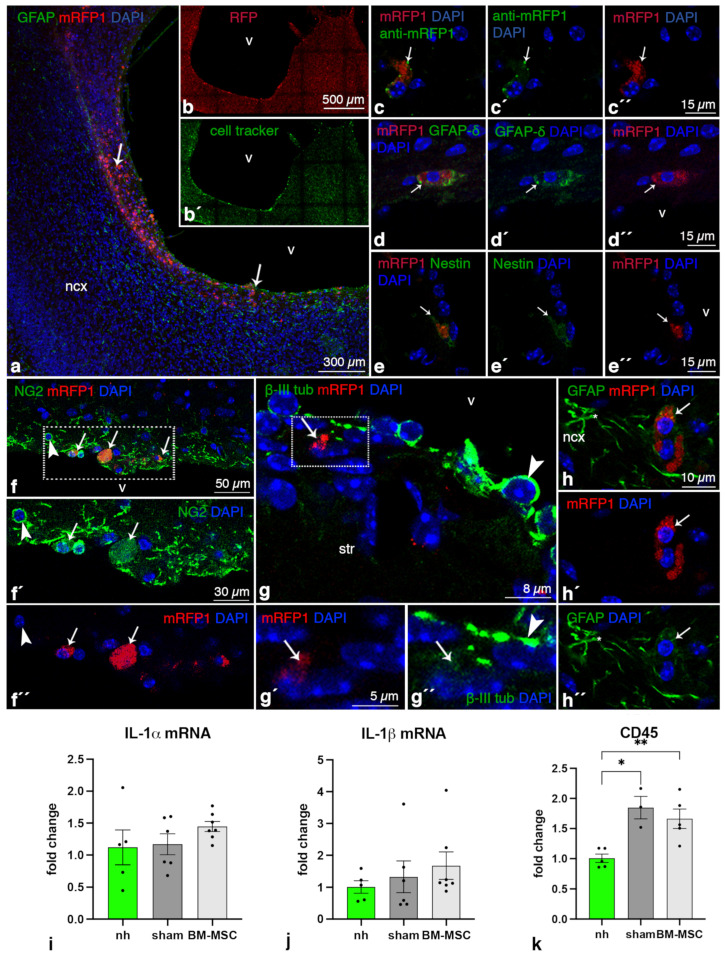
Integration of BM-MSCs into the periventricular cerebral tissue of hydrocephalic hyh mice. (**a**) Wall of the neocortical (ncx) lateral ventricle (v) of a hydrocephalic hyh mouse at P18; administered BM-MSCs (white arrows, mRFP1 fluorescence in red) at P4. BM-MSCs are located between periventricular reactive astrocytes (GFAP, green). (**b**,**b′**) Separated channels showing fluorescence of the green cell tracker and mRFP1 (red) in the transplanted BM-MSCs. (**c**,**c′**,**c″**) Labeling of BM-MSCs (arrow, mRFP1 fluorescence in red) with an antibody against mRFP1 (green, white arrow). Expression of (**d**,**d′**,**d″**) δGFAP (green), (**e**,**e′**,**e″**) nestin (green), (**f**–**f″**) NG2 (green), (**g**–**g″**) βIII-tubulin (green), and (**h**–**h″**) GFAP (green) in BM-MSCs (mRFP1, fluorescence in red, arrows). (**f’**,**f″**,**g′**,**g″**) are separated channels for fluorescence of the cells indicated in the framed area in **f** and **g**, respectively. The BM-MSCs present a weak immunoreaction (arrows) compared to NG2 cells and reactive astrocytes of the host tissue (arrowheads in (**f**,**f′**), arrows in (**h**–**h″**)). Magnifications are the same in the different separated channels. All images are from immunofluorescence in vibratome sections. mRNA levels of the interleukins (**i**) IL-1α and (**j**) IL-1β, and (**k**) CD45. Means ± SEM are shown (nonhydrocephalic mice, nh; = 5; hyh hydrocephalic sham controls, sham, n = 6, n = 4 for CD45; hyh hydrocephalic treated with BM-MSCs, BM-MSC, n = 7, n = 5 for CD45). * *p* < 0.05, ** *p* < 0.01; Wilcoxon–Mann–Whitney test. Abbreviations: str, striatum.

BM-MSCs were found in the periventricular parenchyma containing the astrocyte reaction in both lateral ventricles of hydrocephalic hyh mice 14 days after their transplantation (Figure 1a,b). Transplanted BM-MSCs were detected by their red mRPF1 fluorescence (Figure 1a), with green cell tracker colabeling (previously applied in vitro, Figure 1b), and after the immunodetection of mRPF1 with an appropriate antibody (Figure 1c). Experiments with longer survival times were not designed because hyh mice with severe hydrocephalus usually do not survive more than three weeks [31].

### 2.2. BM-MSCs Do Not Transdifferentiate into Neural Cells after Transplantation

The expression of some stem cell markers was studied to confirm that BM-MSCs did not transdifferentiate after transplantation. Transplanted BM-MSCs maintained the expression of nestin (detected with the antibody Rat-401), NG2, βIII-tubulin, δGFAP, and αGFAP (Figure 1d–h) in the same way as they did in vitro (for details, see [17]) indicating no transdifferentiation. NG2, βIII-tubulin, and αGFAP immunoreactions in the transplanted BM-MSCs were weak compared to the corresponding labeling in oligodendrocyte progenitors, neuroblasts, or astrocytes, respectively.

### 2.3. BM-MSCs Do Not Induce an Inflammatory Reaction after Transplantation

BM-MSC transplantation did not apparently induce inflammatory or microglial responses, as IL-1α, IL-1β (proinflammatory interleukins), and CD45 (activated microglia marker and immune cell marker) levels in the cerebral tissue were similar between the BM-MSC-transplanted and sham-injected hydrocephalic hyh mice (Figure 1i–k). Furthermore, activated CD45+ microglia or neutrophils were not detected in brain sections near the transplanted BM-MSCs.

### 2.4. Higher Levels of NGF, VEGF, HIF1α and TGFβ1 Are Present in the Cerebral Tissue of Hydrocephalic Transplanted Mice

**Figure 2:**
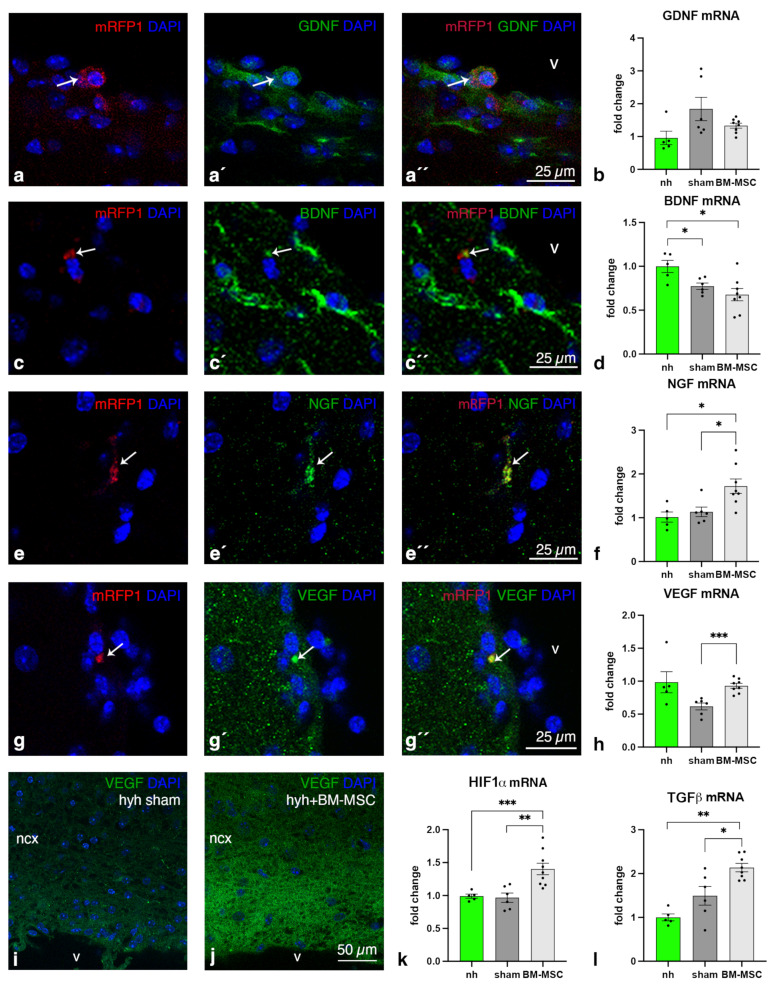
Neuroprotective factors in hydrocephalic hyh mice treated with BM-MSCs. Immunofluorescence images detecting (**a,a′**,**a″**) GDNF, (**c**,**c′**,**c″**) BDNF, (**e**,**e′**,**e″**) NGF, and (**g**,**g′**,**g″**) VEGF (green) in BM-MSCs (arrows) in the neocortex (ncx) from a hydrocephalic hyh mouse transplanted with BM-MSCs at P18. Separated and merged channels for fluorescence are shown. Fluorescence of mRFP1 is in red. (**b**,**d**,**f**,**h**) mRNA levels of GDNF, BDNF, NGF, and VEGF in the nonhydrocephalic mice (nh, n = 5), sham-injected hydrocephalic hyh mice (sham, n = 7), and BM-MSC-transplanted (BM-MSC, n = 9) hydrocephalic hyh mice at P18. (**i**,**j**) Immunofluorescence for VEGF in cerebral sections of the sham-injected and BM-MSC-transplanted hydrocephalic hyh mice. (**k**,**l**) mRNA levels of HIF1α and TGFβ1 in the nonhydrocephalic mice (nh, n = 5), sham-injected hydrocephalic hyh mice (sham, n = 6), and BM-MSC-transplanted (BM-MSC, n = 8) hydrocephalic hyh mice at P18. Means ± SEM are shown. * *p* < 0.05; ** *p* < 0.02; *** *p* < 0.01; Wilcoxon–Mann–Whitney test. Nuclear staining in blue with DAPI. Abbreviations: ncx, neocortex; v, lateral ventricle lumen.

The expression of several neuroprotective factors was studied to detect any improvement after stem cell therapy. Transplanted BM-MSCs were detected to express glial cell-derived neurotrophic factor (GDNF) and brain-derived neurotrophic factor (BDNF) (Figure 2a,c). However, quantification of mRNA in the overall cerebral tissue did not reveal differences between the hyh mice with BM-MSCs and the sham-injected controls (Figure 2b,d). Other growth factors, nerve growth factor (NGF) and vascular endothelial growth factor (VEGF) were detected in transplanted BM-MSCs (Figure 2e,g). In contrast to GDNF and BDNF, the cerebral tissue of the hyh mice injected with BM-MSCs contained higher levels of NGF and VEGF than that of the sham-injected hyh mice (Figure 2f–j). Similarly, VEGF and hypoxia-inducible factor 1 alpha (HIF1α) were detected in the transplanted BM-MSCs, and their mRNA levels in the cerebral tissue were increased after treatment (Figure 2h–k). Because VEGF expression can be induced by hypoxia-inducible factor 1α (HIF1α) [32] and both factors could lead to angiogenesis [33], the area of blood vessels in cerebral sections was quantified, but no differences were found (Figure S1). Finally, mRNA levels in the cerebral tissue of transforming growth factor β1 (TGFβ1), a factor implicated in astrocyte physiology [34], were also increased in the hyh-treated mice compared with the sham controls and normal mice (Figure 2l), indicating a possible effect of transplantation on the astrocyte reaction.

### 2.5. BM-MSC Treatment Induces Changes in the Proteome Related to Neural Development

**Figure 3:**
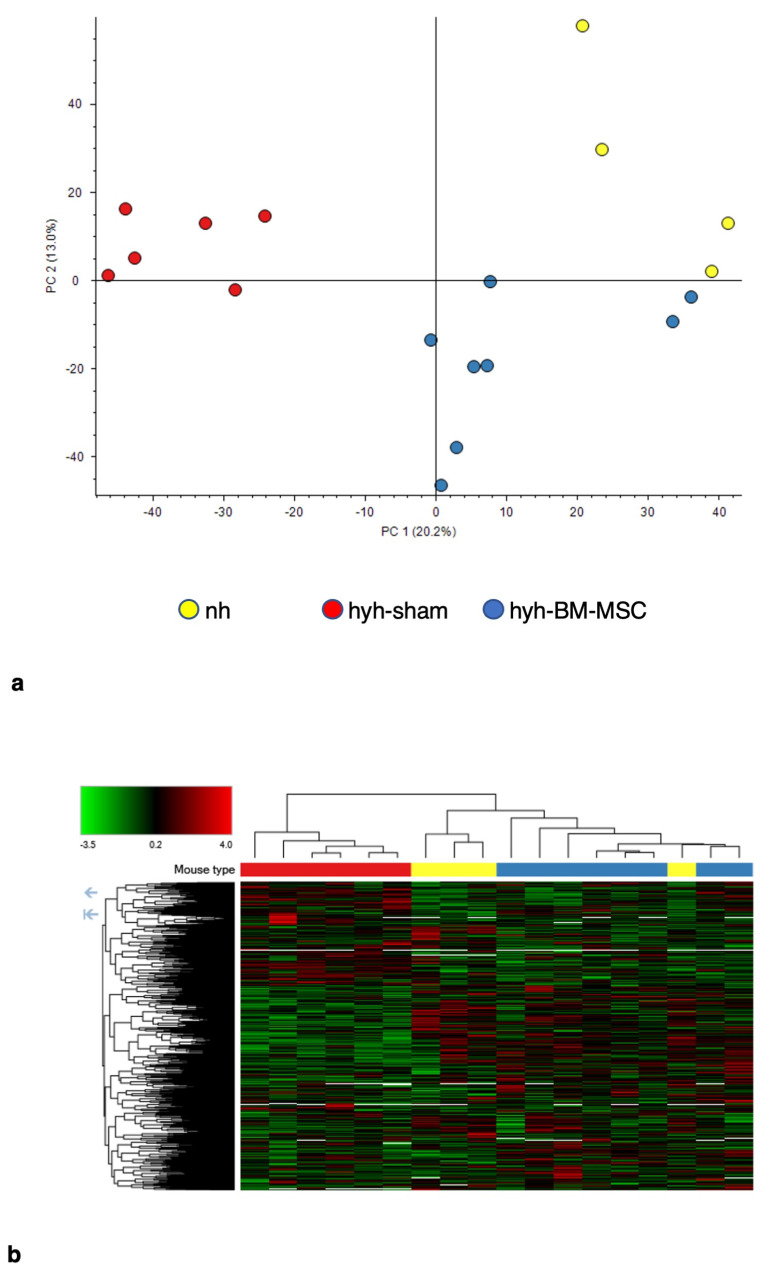
Differential expression of proteins between BM-MSC-transplanted and sham-injected hydrocephalic hyh mice. (**a**) Principal component analysis of the hyh mice treated with BM-MSCs (hyh-BM-MSC, n = 8), hyh mice sham-injected (hyh-sham, n = 6), and nonhydrocephalic normal mice (nh, n = 4). (**b**) Heatmaps and clusters of the three groups of mice.

**Figure 4:**
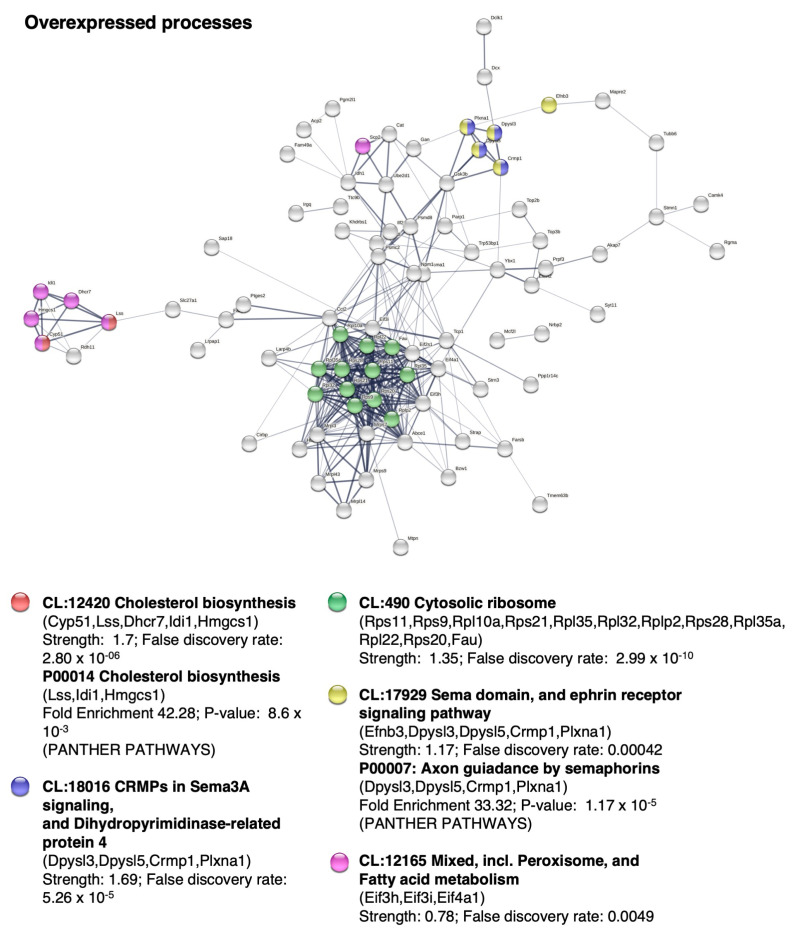
Gene Ontology and STRING analysis of the overexpressed proteins between BM-MSC-transplanted and sham-injected hydrocephalic hyh mice. Analysis and representation with STRING comparing the BM-MSC-transplanted (n = 8) hydrocephalic hyh mice with the sham-injected hydrocephalic hyh mice (n = 6) at P18. The analysis resulted in the categorization of proteins into various groups based on biological processes (GO, GO-Slim biological processes in PANTHER; P, PANTHER PATHWAYS) and local network clusters (CL, STRING). The percentage of genes against the total number of genes in the list is indicated. Reference gene expression corresponds to proteins found in the sham-injected mice. In PANTHER, fold enrichment represents the number of genes whose expression changed over the expected expression. A binomial test with Bonferroni correction for multiple testing with *p* < 0.05 was applied. In STRING, strength represents Log10 (observed/expected). This measure describes how large the enrichment effect is. It is the ratio between (i) the number of proteins in the network that are annotated with a term and (ii) the number of proteins that are expected to be annotated with this term in a random network of the same size. False discovery rate describes how significant the enrichment is. The *p* values corrected for multiple testing within each category using the Benjamini–Hochberg procedure are shown.

**Figure 5:**
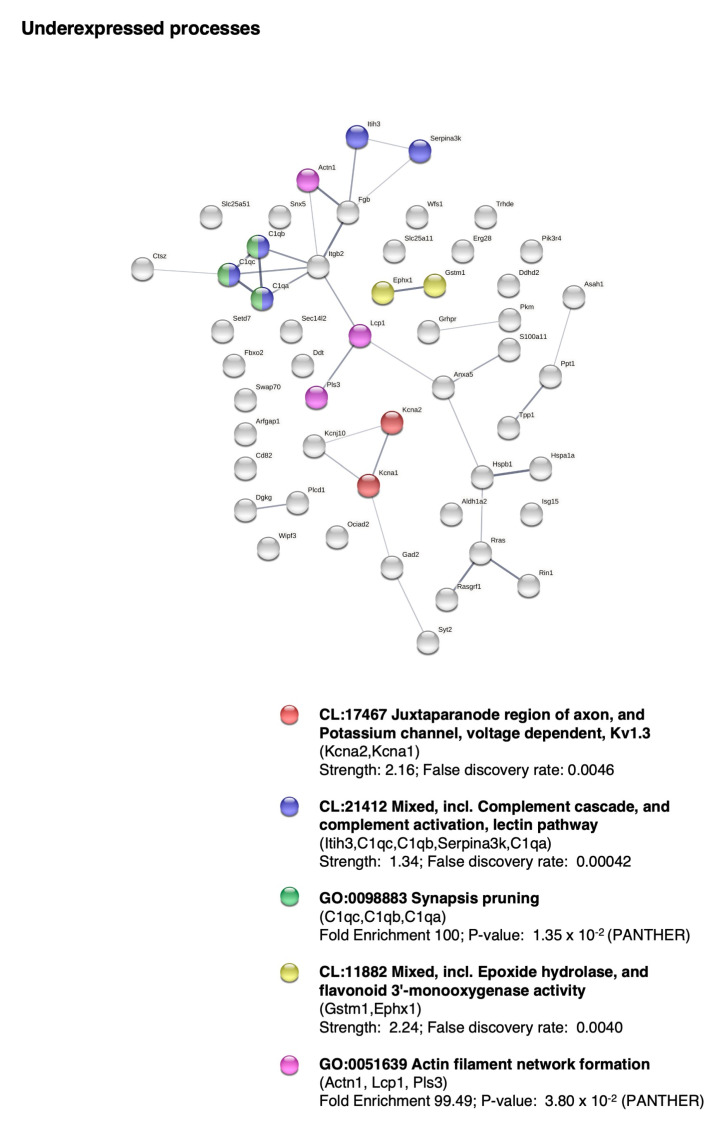
Gene Ontology and STRING analysis of the underexpressed proteins between BM-MSC-transplanted and sham-injected hydrocephalic hyh mice. Analysis and representation with STRING comparing the BM-MSC-transplanted (n = 8) hydrocephalic hyh mice with the sham-injected hydrocephalic hyh mice (n = 6) at P18. For more details, see the legend in Figure 4.

To test if there was an improvement after treatment and to explore possible molecular/cellular pathways, we analyzed the protein expression profile by mass spectrometry. The principal component analysis and the heatmap of protein expression differentiated the three groups of mice, confirming that BM-MSCs had a significant influence on nervous tissue development (Figure 3). The protein expression profile of the hyh mice treated with BM-MSCs was more similar to that of the nonhydrocephalic mice than to that of the sham control mice (Figure 3a), suggesting amelioration after treatment. Cholesterol biosynthesis (CL:12420 and P00014 pathways) and axonal guidance signaling (CL:18016, CRMPs in Sema3A signaling and dihydropyrimidinase-related protein 4; sema domain; and CL:17929, ephrin receptor signaling pathway, P00007; axon guidance by semaphorins) were the main enhanced cellular processes after BM-MSC treatment (Figure 4, Table 1). In addition, proteins of the cytosolic ribosome pathway (CL:490) were overexpressed. In contrast, synapsis pruning (CL:21412 mixed, including complement cascade, and complement activation, lectin pathway; GO:0098883, synapsis pruning) was detected as one of the inhibited cell processes (Figure 5, Table 2).

### 2.6. BM-MSC Treatment Induces the Proliferation of Astrocytes Present in the Periventricular White Matter

**Figure 6:**
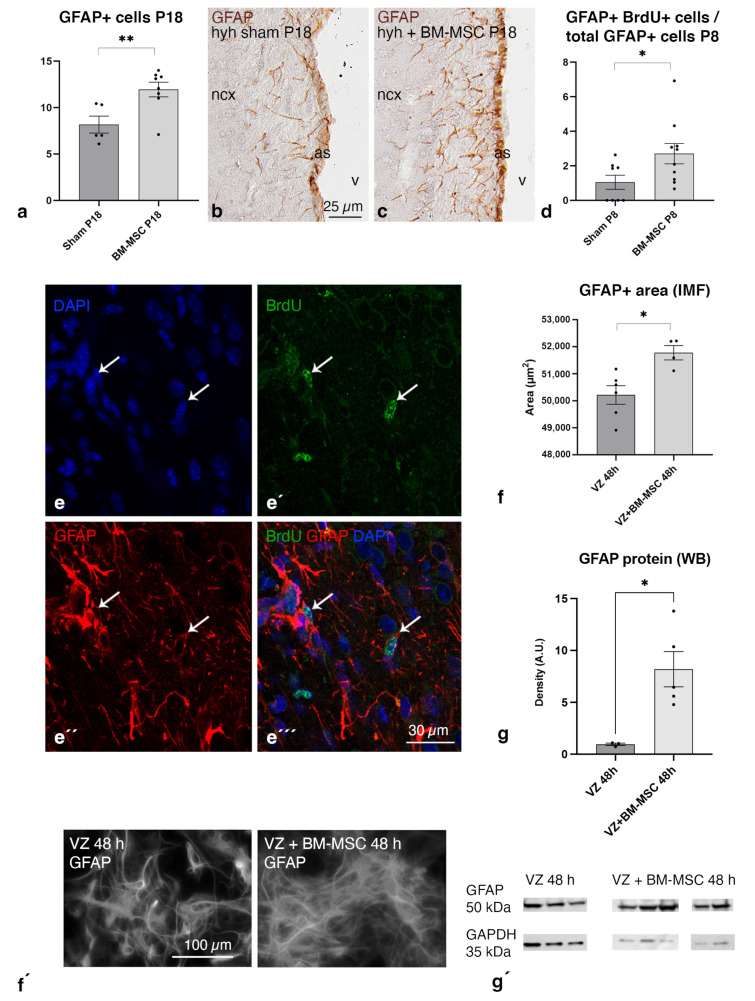
Astroglial stimulation after BM-MSC transplantation in hydrocephalic hyh mice. Density of (**a**) GFAP+ cells in the sham-injected (sham, n = 5) and BM-MSC-transplanted (*BM-MSC*, n = 8) hydrocephalic hyh mice at P18. Representative images used for quantification with GFAP labeling in paraffin sections of the neocortex (ncx) from (**b**) a hydrocephalic hyh mouse sham-injected (*sham*) and (**c**) a hydrocephalic hyh mouse transplanted with BM-MSCs (BM-MSC). Magnifications are the same. The astrocyte reaction at the interphase between the brain parenchyma (as, see [27]) and the ventricle (v) was excluded from the quantification. (**d**) Densities of GFAP+ BrdU+ cells in the sham-injected (sham, n = 8) and BM-MSC-transplanted (BM-MSC, n = 10) hydrocephalic hyh mice at P8. Immunofluorescence in frozen sections of (**e–e‴**) the sham-injected hydrocephalic hyh mice at P8 against BrdU (green) and GFAP (red) showing colabeling in some astrocytes (white arrows). Nuclear staining with DAPI in blue. Means and SEM are shown; * *p* < 0.05, ** *p* < 0.02; Student’s *t* test. Area occupied by GFAP+ astrocytes (**f**) in the plates (VZ plates, n = 5; VZ + BM-MSC plates, n = 4) and density of GFAP+ bands (**g**) (*A. U.*, arbitrary units) in the protein extracts detected with immunoblotting (VZ wells, n = 3; VZ + BM-MSC wells, n = 5) in the plates of the in vitro experiments of ventricular zone (VZ) cells 48 h after coculture with BM-MSCs. Means ± SEM are represented; * *p* < 0.05; ** *p* < 0.02; Mann–Whitney test. Representative images used for quantification are shown below in (**f′**,**g′**).

Due to the relevance of the astrocytic reaction in hydrocephalus, the quantification of astrocytes (GFAP-positive cells) was carried out in brain sections 4 and 14 days after the treatment. BrdU was administered one day before sacrifice to quantify the astrocytic proliferation rate at P8 when the astrocyte reaction develops. A higher density of reactive astrocytes was detected in the periventricular white matter of the hyh mice treated with BM-MSCs compared to sham-injected hyh mice after 14 days of treatment (Figure 6a–c). Accordingly, four days after treatment, at P8, there was a higher number of BrdU+ astrocytes (GFAP+) in the treated mice compared to the sham controls (Figure 6d,e). The analysis of protein overexpression in the cerebral tissue at P8, when the astrocyte reaction initiates in the hyh mouse [25], revealed significant changes in reactome pathways (MMU) involved in protein biosynthesis and ribosomes, consistent with the existence of cell proliferation (Figures S2 and S3). 

### 2.7. BM-MSCs Stimulate the Astrocyte Proliferation in the Ventricular Zone In Vitro

Based on the in vivo results showing astrocyte proliferation after treatment, we decided to perform in vitro studies to confirm that BM-MSCs can stimulate astrocyte proliferation in the ventricular zone. We employed a murine in vitro model to mimic the ventricular zone of neonates, which contains astrocytes, neural stem cells, and multiciliated ependyma [35]. The in vitro experimental coculture of ventricular zone cells with BM-MSCs confirmed the stimulation of astrocyte proliferation through an increase in the GFAP+ area and higher levels of GFAP in the protein extracts (Figure 6f,g).

### 2.8. Upregulation of AQP4 Expression in the Periventricular White Matter of Hydrocephalic Treated Hyh Mice

**Figure 7:**
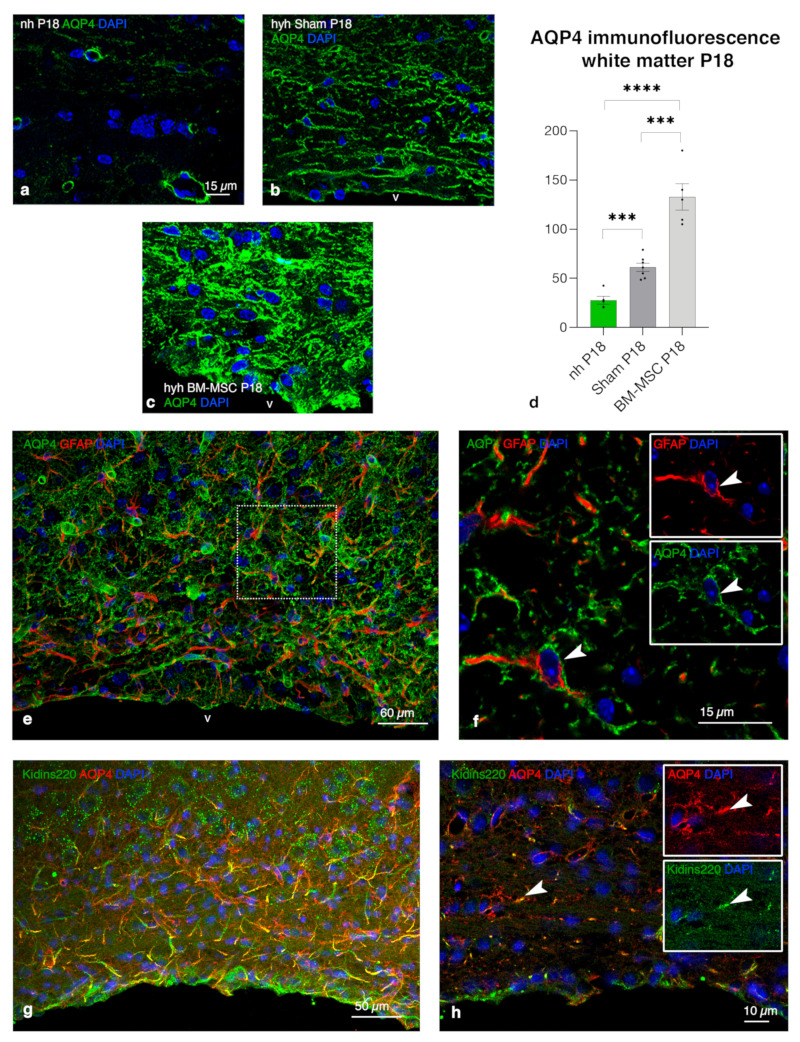
Increased expression of AQP4 and Kidins220 colocalization in the periventricular white matter of hydrocephalic mice treated with BM-MSCs at P18. (**a**–**d**) Immunofluorescence intensity for AQP4 in the nonhydrocephalic mice (nh, n = 4), in hydrocephalic hyh mice treated with BM-MSCs (n = 5) and hyh mice used as sham controls (n = 7). Means ± SEM are shown. *** *p* < 0.01, **** *p* < 0.0005; Student’s *t* test. (**e**,**f**) Presence of AQP4 (green) in cell bodies and processes of reactive astrocytes (GFAP in red, arrowheads). The detail framed in (**e**) is shown in (**f**). (**g**,**h**) Colocalization of AQP4 (red) and Kidins220 (green) in reactive astrocytes (arrowheads). Inserts in (**f**,**h**) represent separated channels for GFAP, AQP4, or Kidins220. Nuclear staining with DAPI in blue. Abbreviations: v, lateral ventricle lumen.

Finally, to uncover a possible neuroprotective role of astrocytes after treatment, AQP4 expression was analyzed. The expression of AQP4 was studied by immunofluorescence in tissue sections of the hyh mice treated with BM-MSCs, sham controls and nonhydrocephalic mice. According to the proliferation of the astrocyte reaction in the treated hyh mice, AQP4 immunostaining was increased in the periventricular white matter (Figure 7a–d). In nonhydrocephalic mice, AQP4 expression was only detectable in astrocyte perivascular endfeet; but in reactive astrocytes in the hyh hydrocephalic treated and sham control mice, AQP4 was also detected in their cell bodies and processes (Figure 7e,f). Reactive periventricular astrocytes also presented the same pattern of overexpression in cell bodies and processes of Kidinss220 (Kinase D interacting substrate of 220 kDa, a transmembrane protein that plays a key role in the regulation of AQP4 content [36]), highly colocalizing with AQP4 (Figure 7g,h).

## 3. Discussion

Herein, we show that transplanted BM-MSCs integrate into the reactive astrocyte in the periventricular walls of hydrocephalic hyh mice, where they could survive for 14 days. BM-MSCs did not transdifferentiate or produce an inflammatory reaction. Fourteen days post-treatment, the neocortical tissue conditions improved by an increase in the levels of the neuroprotective factors NGF, VEGF, HIF1α, and TGFβ1; an overexpression of proteins involved in axonal growth and cholesterol synthesis indicative of a neuro-regenerative environment; and, importantly, the proliferation of the astrocytes overexpressing the potential neuroprotective AQP4.

### 3.1. Transplanted BM-MSC Integrated without Rejection into the Periventricular Astrocyte Reaction

Transplanted BM-MSCs were found located in the most damaged cerebral areas, the periventricular white matter and the ventricular/subventricular zone [6,25,37,38,39], where they could be attracted by chemokines or TNFα [18,21], the latter produced by periventricular reactive astrocytes in hyh mice [40]. Our results suggest that BM-MSCs do not transdifferentiate into any neural cell type in the times studied. Integrated BM-MSCs maintained the expression of nestin, δGFAP, GFAP iba1, NG2, and βIII-tubulin, in the same way as they did in vitro before transplantation. These results are in agreement with our previous study in hyh mice at P20, when hydrocephalus is already severe, and BM-MSCs were detected four days later [17]. Furthermore, our data on interleukin and CD45 levels support an absence of immunological/microglial reaction. BM-MSCs can produce factors that affect the maturation and function of immune cells, suppressing innate and adaptive immunity [18]. 

### 3.2. BM-MSCs Stimulate Proliferation of Astrocytes Overexpressing AQP4 in the Periventricular Edema

The present investigation has proven the stimulation of developing periventricular astrocyte reaction overexpressing AQP4 after BM-MSC administration, and the results were corroborated using an in vitro model. In the case of the hydrocephalic hyh mouse, the astrocytic reaction originates during the first week of postnatal life [25,41]. Thus, transplanted BM-MSCs could affect astrocytes at the earlier stages of the reaction (transplanted at P4). BM-MSCs can increase astrocyte proliferation [42,43] and activate astrocytes through the production of GDNF (detected in the present study in the transplanted BM-MSCs), which could together contribute to a functional improvement in brain ischemia [44] present in hydrocephalus with high ICP. However, controversy regarding the detrimental neuroprotective effect of the astrocyte reactions should be considered [45,46]. Furthermore, in the white matter, the pro-inflammatory (A1) or anti-inflammatory (A2) reactive astrocyte phenotypes after BM-MSC treatment will need further analyses. In the case of hyh mouse hydrocephalus, evidence indicates that the astrocyte reaction could have a protective effect, where AQP4 can play a relevant role in ependyma-denuded areas [27]. A higher expression of AQP4 has also been described in the ventricular walls of hydrocephalic pediatric cases [10] and in animal models of congenital [27] and experimental hydrocephalus [9,10], where it has been considered to play a protective role [9,13,47]. Accordingly, silencing of AQP4 has been proven to aggravate hydrocephalus [48]. 

### 3.3. An In Vitro Model to Study the Stimulation of Astrocytes by BM-MSCs

One major factor that can limit scientific progress in neurological diseases is a lack of appropriate models. Developing an in vitro model of the ventricular zone has allowed for the study of the mechanisms involved in the ventricular zone function and pathology, including the stimulation of astrocytes [35,49]. It is necessary to model the clinical timeline of ventricular zone development as closely as possible. Our experiments were carried out in the stage of differentiation when cultured cells progress to multiciliated ependyma and astrocytes (day 3 of differentiation). Similar to the in vivo results, the coculture of BM-MSCs with the ventricular zone stimulated astrocyte proliferation. As mentioned above, BM-MSCs have been described to increase astrocyte proliferation in other models through GDNF. Still, the molecular mechanism behind this stimulation will need further analysis in this in vitro model.

### 3.4. Overexpression of AQP4 as a Potential Neuroprotective Effect on the Periventricular Edema

The analysis of the molecular mechanism by which AQP4 can generate an improvement in hydrocephalus can be complex [13]. AQP4 seems to be a crucial player in the change from cytotoxic to vasogenic edema, where the water channel contributes to edema clearance into CSF compartments and veins [50]. Thus, it is likely that AQP4 overexpression in the periventricular areas of treated hyh mice could contribute to edema clearance and, therefore, to tissue improvement. However, another type of edema is present in hydrocephalus with high ICP, known as interstitial transependymal edema, which has a different origin [51,52], and the role of AQP4 still needs to be understood. Nevertheless, it can be supposed that AQP4 overexpression should have functional consequences in hydrocephalic edema [13]. Whether these astrocytes can be directly implicated in the tissue recovery previously described in the hyh mouse after BM-MSC transplantation [17] needs to be addressed. Furthermore, the role that AQP4 plays in the glymphatic circulation is unknown. Therefore, further research will be necessary to unveil the contribution of AQP4 to cerebral water movement after BM-MSC treatment.

### 3.5. BM-MSC Environment Can Play a Neuroprotector Role and Be Implied in AQP4 Upregulation

The functional improvement that a designed stem cell therapy can have in a neurological disease can be caused by the direct effect of the cell engraftment, the immunomodulation by molecular factors secreted by the transplanted cells, [53] or by the stimulation of the host tissue, such as the astrocyte reaction. In our study, the BM-MSC environment may explain the results of the stimulation of the astrocyte reaction, their AQP4 expression, and the presence of a neuroprotector effect in hydrocephalus development. Integrated BM-MSCs expressed the neurotrophic factors GDNF, BDNF, NGF, and VEGF. These factors have been suggested not only to potentiate neuronal survival and reduce apoptosis but also to increase blood supply and functional recovery under ischemic conditions [20,54,55,56,57]. Moreover, the production of these factors can be enhanced in hypoxic brain conditions [58], a state that could be present in the hydrocephalic hyh mice exhibiting a high ICP [26]. 

The increased mRNA levels of VEGF and HIF1α detected in the cerebral tissue of hyh mice transplanted with BM-MSCs may be related to AQP4 overexpression. VEGF and HIF1α have been associated with AQP4 regulation. Several investigations have suggested an interplay between AQP4 and VEGF or that a common mechanism may participate in regulating AQP4 and VEGF expression [59]. Hypoxia can induce the production of both molecules [59,60,61,62]. There is also evidence of functional interactions between AQP4 and HIF1α, the major transcription factor regulating VEGF expression [59]. Then, the increased expression of AQP4 can be linked to the increased levels of VEGF and HIF1α in treated mice. In addition, increased astrocytes in hyh-treated mice could play beneficial roles in regulating angiogenesis and vascular maturation. BM-MSCs and VEGF production can enhance the interaction between astrocyte and brain endothelial cells, promoting vascular stabilization [33,63]. 

### 3.6. Other Neuroprotective Effects of BM-MSCs beyond AQP4 Upregulation

In addition to the effects related to astrocyte reaction and AQP4 expression, the increased levels of NGF and TGFβ1 in the cerebral tissue suggest a beneficial role of BM-MSCs. Higher TGFβ1 levels have been related to the origin of communicating and posthemorrhagic hydrocephalus [64,65,66]. However, in animal models of obstructive hydrocephalus, including the hyh mouse, TGFβ1 expression is decreased or unchanged [40,67]. Astrocytes can release TGFβ1 with an immunoregulatory role after stroke [68] and a neuroprotective function [69]. TGFβ1 can increase NGF expression after central nervous system injury [70]. Higher levels of NGF mRNA after BM-MSC treatment could indicate that it is neuroprotector. NGF has been reported to protect neurons against glutamate excitotoxic damage and ischemia [71], both conditions present in hydrocephalic hyh mice [26]. 

Our protein expression data indicate changes that can be related to neural development or to a neuroregenerative environment. The most significant overexpressed process after BM-MSCs was cholesterol biosynthesis, which is necessary for neuronal differentiation and synaptogenesis [72]. In the brain, cholesterol is mainly present in myelin and is synthesized by oligodendrocytes but at low levels in neurons and astrocytes [72]. Other significantly overexpressed processes are related to well-known mechanisms of axon guidance signaling through semaphorin and ephrin receptors [73,74]. This evidence could indicate a beneficial effect of BM-MSCs on the restoration of the periventricular white matter. As another remarkable effect, BM-MSC treated hyh mice presented an underregulation of complement proteins implicated in synaptic pruning, which involves microglia and astroglia [75]. Accordingly, MSCs from the umbilical cord have been described to affect synaptic plasticity [76].

### 3.7. Limitations of the Study

Injections for the administration of BM-MSCs or saline in the sham controls during the first week of life in hyh mice can influence ICP evolution and other related parameters of pathology [26]. The introduction of a needle can divert intraventricular CSF to other cranial cavities and in a similar way to spontaneous ventriculostomies. The existence of such ventriculostomies can underlie the evolution of the disease in the hyh mouse [31]. Therefore, the analysis of ICP as a parameter to measure improvement after treatment may not be reliable in our mouse model, and it was not considered for evaluation in this study. Thus, proteomics was performed as an alternative analysis of improvement post-treatment. Furthermore, sham-operated hyh mice were selected as the most appropriate controls to discard any possible side effects of the injection since they were administered with the same volume of saline but no cells. Therefore, all the changes detected after treatment were confirmed to be caused by BM-MSC. The hyh mouse model does not allow for long-term testing after transplantation because these animals cannot survive more than three weeks [31].

## 4. Materials and Methods

### 4.1. Experimental Animals

BM-MSCs were obtained from transgenic homozygous mice of both sexes and 25 days of age that express monomeric red fluorescent protein 1 (mRPF1; Tg(GAG-mRPF1)aF1Hadj/J). The following animal groups were used: (1) four-day-old (P4) hyh males and females (B6C3Fe-a/a-hyh/J strain) were used to transplant BM-MSCs (hydrocephalic hyh BM-MSC-injected group) or sterile saline (hydrocephalic hyh sham-injected control group), and were sacrificed 14 days later; (2) eighteen-day-old (P18) males and females nonhydrocephalic littermates were used as another control group (nonhydrocephalic group, nh); and (3) P4 hyh mice were injected with either BM-MSCs or saline and were sacrificed four days later for bromodeoxyuridine (BrdU) and proteome analyses. Hyh and nonhydrocephalic mice were identified by phenotype inspection and genotyping [77]. Nonhydrocephalic mice were used as controls for healthy referent values to compare after BM-MSC treatment. All mice were originally obtained from The Jackson Laboratory (Bar Harbor, ME, USA) and bred in the Animal Experimentation Service of the University of Malaga at 22 °C with a 12:12 light/dark cycle and standard food and water available ad libitum. The design of the experiments, housing, handling, care, and processing of the animals were conducted in accordance with European and Spanish laws (RD53/2013 and 2010/63UE) and following ARRIVE guidelines. According to current legislation, experimental procedures were approved by the Institutional Animal Care and Use Committee of the University of Malaga (CEUMA, Malaga, Spain) and the Regional Government Council (Junta de Andalucía, Sevilla, Spain). 

### 4.2. Bone Marrow-Derived Mesenchymal Stem Cells Isolation, Culture, and Characterization

BM-MSCs from the bone marrow of transgenic mRPF1 mice were obtained and characterized as previously described [17]. Briefly, BM-MSCs were selected according to their plastic adhesion in vitro, their positive expression of CD44, CD73 and CD90, their negative expression of CD34 and CD45, and their proven trilineage differentiation capacity [17]. Dulbecco’s modified Eagle’s medium (DMEM, Sigma-Aldrich, St Louis, MO, USA) containing 1% penicillin/streptomycin (P/S), 0.5% amphotericin B, 6.25% L-glutamine, and 10% fetal bovine serum (FBS, Sigma-Aldrich) was used as the culture medium. After confluence, BM-MSCs at passage one were detached with trypsin/ethylenediaminetetraacetic acid (EDTA; Sigma-Aldrich), centrifuged at 400× *g* for 5 min and resuspended in sterile saline at 5000 cells/µL in 4 µL. 

For confirmation of stem cell therapy viability, red fluorescent BM-MSCs were also labeled with a green cell tracker dye (C2925, Molecular Probes, Thermo Fisher, Waltham, MA, USA) before their transplantation. 

### 4.3. Bone Marrow-Derived Mesenchymal Stem Cell Transplantation

Four-day-old hydrocephalic hyh mice were anesthetized with 4% sevoflurane in 1 l/min of oxygen. BM-MSCs (5000 cells per µL of sterile saline, 20,000 cells in a total volume of 4 µL) were transplanted into the right lateral ventricle with a 26-gauge needle syringe (Hamilton, 701RN, Merck Millipore, Burlington, MA, USA). In addition, a group of hydrocephalic hyh mice were sham-injected with the vehicle solution but no BM-MSCs, following the same procedure and administering the same volume (4 µL total). The vehicle solution for both sham controls and treated mice was sterile saline (0.9% sodium chloride). A 26-gauge needle syringe (Hamilton Syringe 75N, Hamilton, Reno, NV, USA) was free-hand used at coordinates in the middle between the orbit level and the ear and was 1 mm deep. The coordinates were previously assayed with trypan blue injections. A total volume of 4 µL was injected for 30 s. The needle was left for 10 s more before removing. Mice were processed 4 days (at P8, see below in experiments with bromodeoxyuridine labeling) or 14 days after transplantation (at P18).

### 4.4. Immunofluorescence and Immunocytochemistry

Nonhydrocephalic mice (n = 5), hydrocephalic hyh mice transplanted with BM-MSCs (n = 34), and hydrocephalic sham-injected mice (n = 31) were sacrificed under anesthesia with Dolethal (sodium pentobarbital; intraperitoneal administration, 0.2 mg/g body weight; Vétoquinol, Lure, France) and transcardially perfused with either 4% paraformaldehyde diluted in 0.1 M, pH 7.2, phosphate buffer or Bouin’s fixative. Fixed brains were dissected out and postfixed in the same solution for 24 h (paraformaldehyde, 4 °C) or for 48–72 h (Bouin, room temperature). 

Paraformaldehyde-fixed brains were sectioned with a vibratome (70 μm thick sections) or cryoprotected in 30% sucrose to obtain frozen sections (60 μm thick). Vibratome sections were chosen when detection of mRFP1 or cell tracker fluorescence was needed. The vibratome and frozen sections were processed with a free-floating section staining protocol for immunofluorescence using unconjugated primary antibodies (Table 3). Secondary antibodies conjugated with Alexa Fluor 488 or Alexa Fluor 568 (Thermo Fisher, Waltham, MA, USA) were used. Nuclear staining was performed with 4′,6-diamidino-2-phenylindole dihydrochloride (DAPI). 

Bouin-fixed brains were embedded in paraffin to obtain 10 µm thick seriated sections. The sections were hydrated, and heat-induced antigen retrieval in citrate (50 mM, pH 6.0) was carried out. Then, the sections were incubated with anti-GFAP (Table 3), followed by biotinylated secondary antibodies (Dako, Glostrup, Denmark), ExtrAvidin-peroxidase (Sigma-Aldrich), and 3,3′-diaminobenzidine tetrahydrochloride (DAB, Sigma-Aldrich) for histochemical detection. Some of the seriated sections were stained with hematoxylin–eosin.

In all the immunofluorescence/immunohistochemical procedures, the antibodies were diluted in phosphate saline buffer (PBS), 0.1 M, pH 7.2, containing 0.05% Triton X-100 (Sigma), 0.01% sodium azide, bovine albumin, and appropriate normal sera. Primary antibody incubations were performed for 18 h at 22 °C or 72 h at 4 °C. Secondary antibody and ExtrAvidin incubations were performed for 1 h at 22 °C. For negative controls, primary antibodies were not added. 

### 4.5. Bromodeoxyuridine Labeling

For labeling of cells undergoing proliferation, a single dose of bromodeoxyuridine (BrdU, Sigma-Aldrich, 100 mg/kg) was intraperitoneally administered at P7 in hydrocephalic hyh mice transplanted with BM-MSCs at P4 (n = 10) and hydrocephalic sham-injected mice at P4 (n = 8). Mice were sacrificed 24 h later (P8). BrdU was detected by immunofluorescence as described above, where sections were pretreated with 2 N HCl for 15 min at 37 °C.

### 4.6. In Vitro Assay

Neuroepithelial cells from the ventricular zone in differentiation were harvested from 3-day-old wild-type mice (C57BL/6) and cultured as described by Castaneyra-Ruiz et al. [35]. At confluence, cells were plated on 24-well plates, previously treated with poly-L-lysine (P4707, Sigma-Aldrich) for 1 h at 37 °C, with differentiation media to induce their differentiation into ependymal cells. After three days of differentiation, BM-MSCs were added for 48 h. Then, the cultures were rinsed with PBS, and Western blotting and immunofluorescence were performed. 

Total protein was extracted using 60 µL RIPA buffer (9806 Cell Signaling Technologies, Danvers, MA, USA) with a protease inhibitor cocktail (P8340, Sigma-Aldrich) for 10 min at 4 °C and centrifuged at 8000× *g* for 10 min at 4 °C. Proteins from the supernatant were separated, and each 30 µL sample was run on an SDS-polyacrylamide gel (4–12% polyacrylamide) electrophoresis and transferred to a 0.2 µm nitrocellulose membrane (IB23001, Thermo Fisher) in an iBlot 2 Dry Blotting System (Thermo Fisher) as previously described [35,49]. Then, samples were incubated with primary antibodies (Table 3) and anti-rabbit HRP-conjugated secondary antibody. Immunoreactivity was visualized with a chemiluminescent substrate (ECL, Cell Signaling, Danvers, MA, USA) and imaged using ChemiDoc XRS+ (Bio-Rad, Hercules, CA, USA). GAPDH was used as the protein loading control, and BM-MSCs and ventricular zone cell without BM-MSCs were used as control conditions. The relative level of GFAP was normalized to GAPDH in each case.

For immunofluorescence quantification, ventricular zone cells and BM-MSCs were plated on coverslips treated with poly-L-lysine in 24-well plates. After PBS rinses, the cells were fixed with 4% paraformaldehyde in PBS, pH 7.4, for 5 min. After fixation, the wells were washed with PBS and permeabilized with 5% bovine serum albumin in PBS containing 1% Triton X-100 for 1 h. Then, an antibody against GFAP conjugated with Cy3 (Table 3) was used to detect astrocytes after incubation for 18 h at 22 °C. Nuclear staining was obtained with DAPI. The coverslips were mounted on slides with fluorescent mounting medium (SouthernBiotech, Birmingham, AL, USA). Pictures were taken under a confocal microscope as described below. The percentage of the area occupied by astrocytes (GFAP+ cells) was quantified in 2 fields per well using ImageJ Fiji [78] software (n = 5 wells). As controls, ventricular zone cells with no BM-MSCs were used (n = 6 wells).

### 4.7. Real-Time PCR

RT-PCR analysis was performed in cerebral samples obtained from mice at P18 (nonhydrocephalic mice, n = 5; hydrocephalic hyh mice transplanted with BM-MSCs at P4, n = 9, and hydrocephalic hyh sham-injected mice at P4, n = 10). Mice were sacrificed by cervical dislocation, and the neocortex was quickly dissected out under cold conditions, frozen with dry ice, and stored at −80 °C. Total RNA from the neocortex was extracted with Tripure Isolation Reagent (Roche, Basel, Switzerland). Contaminating DNA was removed using DNase (Sigma-Aldrich). Three micrograms of total RNA was reverse transcribed with a High-Capacity cDNA Reverse Transcription Kit (4374967; Applied Biosystems, Foster City, CA, USA). Forty nanograms of cDNA was mixed with Eagle Taq Master Mix (Sigma-Aldrich) and TaqMan Gene Expression assay probes (CD45, Mm01293577_m1; GAPDH Mm99999915_g1, BDNF, Mm04230607_s1; GDNF, Mm00599849_m1; IL-1α, Mm01336161_m1; IL-1β, Mm00434228_m1; NGF, Mm00443039_m1; TGFβ1, Mm01227699_m1; VEGF, Mm00437306) from Applied Biosystems. Quantification was performed with an ABI Prism 7000 Sequence Detector System (Applied Biosystems). ΔCt values represent glyceraldehyde-3-phosphate dehydrogenase (GAPDH) normalized expression levels and nonhydrocephalic mice as a control condition. The results were expressed using the comparative double-delta Ct method (2^−ΔΔCt^). The slopes of the curves indicated optimal PCR conditions (slopes of 3.2–3.4).

### 4.8. Mass Spectrometry Analysis of Proteins

Two different analyses were carried out to quantify the expression of proteins in two separate analyses. In the first experiment, nonhydrocephalic wild-type mice (n = 6), hydrocephalic hyh mice transplanted at P4 with BM-MSCs (n = 8), and hydrocephalic hyh sham-injected mice (n = 5) were sacrificed 14 days later (at P18). In a second analysis, hydrocephalic hyh mice transplanted at P4 with BM-MSCs (n = 5) and hydrocephalic hyh sham-injected mice at P4 (n = 5) were sacrificed 4 days later (at P8). The following procedure was the same. After cervical dislocation, the cerebral tissue was quickly dissected out under cold conditions, frozen with dry ice, and stored at −80 °C. Quadrupole-orbitrap nano-HPLC-ESI-MS/MS was used for peptide analysis of samples from mice of the different experimental groups. Gel-assisted proteolysis was performed for the proteins entrapped in a polyacrylamide gel matrix. DigestPro MSI (INTAVIS Bioanalytical Instruments AG, Cologne, Germany) was used for protein digestion and sample preparation. Peptides were purified and concentrated using C18 ZipTip (Merck Millipore) according to the manufacturer’s instructions. Samples were injected into an Easy nLC 1200 UHPLC system coupled to a Q Exactive™ HF-X Hybrid Quadrupole-Orbitrap Mass Spectrometer (Thermo Fisher). Data were acquired using Tune 2.9 and Xcalibur 4.1.31.9 (Thermo Fisher) and Swiss-Prot database was used to identify *Mus musculus* proteins, which were analyzed using Proteome Discoverer 2.2 (Thermo Fisher) and the tandem mass spectrometry data analysis program SEQUEST. The false discovery rate (FDR) was calculated using Percolator. For label-free quantification, the Minora feature detector in Proteome Discoverer 2.2 (Thermo Fisher) was used. 

### 4.9. Image Analysis and Quantification

Immunofluorescence images were obtained with a Leica SP8 laser confocal microscope (Leica, Wetzlar, Germany) using a hybrid sensor (HyD) and a Zeiss LSM 880 Airyscan Two-Photon Confocal Microscope (Zeiss, Oberkochen, Germany). Bright-field micrographs were obtained with a Nikon microscope (Nikon, Tokyo, Japan).

For each experiment, immunofluorescence images were obtained in batches using the same settings. Figures were composed applying the same minimal changes in brightness and contrast. 

Quantification of the images was carried out on the original micrographs. Frozen sections were used to quantify the density of BrdU+ GFAP+ cells in immunofluorescence images. They were calculated in the parietal cortex white matter in 3 fields per section for each animal (1 µm thick confocal plane). 

Paraffin sections were used to calculate the density of GFAP+ cells (cells/area) in the whole periventricular areas (10 µm thick sections) in 10 fields per animal. The rate of GFAP+ astrocytes expressing BrdU was calculated in 10 fields per section (10 µm thickness) per animal in the white matter of the parietal cortex. Finally, the density of the blood vessel area (blood vessel area/total area) was calculated in the parietal cortex of paraffin hematoxylin–eosin-stained sections. This density was quantified by establishing an automatic threshold to capture all the blood vessels of the field using ImageJ [79]. The AQP4 immunofluorescence reaction was quantified in the periventricular white matter in frozen sections and calculated in 3 fields per section/animal (1 µm thick confocal plane) using the same autonomous settings in ImageJ software v1.53k.

### 4.10. Statistics

Statistical analyses were performed using KaleidaGraph v4.1.1 (Synergy Software,, Reading, PA, USA) and GraphPad 9.2.0 (GraphPad Software, San Diego, CA, USA). The required sample size was estimated from quantifications in a preliminary similar study [17]. To achieve 80% power with a 5% significance level, the required sample size was estimated using mean differences and pooled standard deviations from preliminary AQP4 tissue quantifications. The number of animals per group is indicated in the figure captions. Animals were numbered without indication of the group, and researchers were blinded to the treatment groups during all experiments. Values are reported in the figures as the mean with standard error of the mean (SEM). First, normality was assessed with Shapiro–Wilk and D’Agostino’s K-squared tests. When samples did not distribute normally, the Wilcoxon–Mann-Whitney test was applied for hypothesis testing (nonparametric analysis). Samples distributed normally were analyzed with Student’s *t* test (parametric analysis). The applied tests are indicated in the figure captions. When the *F* probability from Student’s *t* test was < 0.05, the variance was considered unequal. *p* < 0.05 based on both tests was considered statistically significant. For immunofluorescence/immunohistochemistry, all tissue and in vitro immunostainings and quantifications were repeated twice. AQP4 immunostaining and tissue quantification were repeated thrice, obtaining the same results in all replicates. Replies for immunostaining quantification were made in new sections from the same experimental animals. In the case of in vitro quantification, the replies were performed in new experiments.

## 5. Conclusions

Transplanted BM-MSCs can integrate into the ventricle walls of hydrocephalic hyh mice during hydrocephalus development, remaining undifferentiated and without rejection. In the injured periventricular white matter, BM-MSCs stimulate the development of the astrocyte reaction with AQP4 overexpression, which can be associated with a neuroprotective effect. Further research will be needed to investigate possible neuroprotective mechanisms against hydrocephalus for such AQP4 overexpression.

## Figures and Tables

**Table 1:** Upregulation of proteins associated with the overexpressed pathways in hyh mice 14 days after BM-MSC treatment at P4. Sum PEP score corresponds to the score calculated based on the posterior error probability (PEP) values of the peptide spectrum matches (PSM). Sum PEP score indicates the probability that an observed PSM is incorrect. Abundance ratio (AR) indicates the relation of the protein expression in BM-MSC-transplanted mice/sham-injected hyh mice.

Protein Name	Sum PEP Score	AR	AR *p* Value
28S ribosomal protein S7. mitochondrial	19.568	1.666	0.008
39S ribosomal protein L3. mitochondrial	9.29	1.598	2.701 × 10^−5^
Amyloid-beta A4 precursor protein-binding family A member 1	59.91	1.691	0.0002
Bisphosphoglycerate mutase	14.636	1.905	0.004
Claudin domain-containing protein 1	14.01	1.907	0.019
Cold-inducible RNA-binding protein	36.025	2.136	0.011
Constitutive coactivator of peroxisome proliferator-activated receptor gamma	8.253	1.749	0.019
Coxsackievirus and adenovirus receptor homolog	110.023	1.743	0.003
DNA topoisomerase 2-beta	98.163	1.557	0.007
DNA topoisomerase 3-beta-1	5.518	1.95	0.012
Dynein light chain Tctex-type 1	17.169	1.734	0.013
Electroneutral sodium bicarbonate exchanger 1	90.13	1.566	0.023
Ephrin-B3	12.103	2.089	0.0002
Fatty acyl-CoA reductase 1	10.939	2.961	0.002
Gigaxonin (OS = *Mus musculus*)	13.586	1.506	0.012
GPI inositol-deacylase	68.486	1.318	0.008
Guanine nucleotide exchange factor DBS	6.267	2.914	0.028
Interferon-inducible double-stranded RNA-dependent protein kinase activator A	44.187	1.548	0.003
Isoform 2 of peroxisomal acyl-coenzyme A oxidase 1	149.85	1.749	0.001
KIF1-binding protein	88.274	1.865	0.002
Kinesin-like protein KIF21B	169.817	2.724	0.0002
La-related protein 4B	9.525	1.557	0.019
MARCKS-related protein	58.881	1.753	0.001
Mitofusin-1	6.777	1.538	0.034
Neuron navigator 1	245.817	1.6	0.003
Neuronal migration protein doublecortin	63.588	2.29	0.0005
Nuclear autoantigenic sperm protein	20.927	1.73	0.016
Nuclease-sensitive element-binding protein 1	137.43	1.675	0.037
Poly [ADP-ribose] polymerase 1	24.147	2.214	0.003
Protein phosphatase 1 regulatory subunit 14C	12.2	1.893	0.012
Shootin-1	39.062	1.601	0.005
Synaptotagmin-11	15.866	1.588	0.016
Tetratricopeptide repeat protein 9B	29.372	1.846	0.035
Transcription factor BTF3	15.06	1.679	0.012
Translation initiation factor eIF-2B subunit delta	27.65	1.557	0.023
Tubulin beta-6 chain	296.149	2.272	0.001
U4/U6 small nuclear ribonucleoprotein Prp3	11.174	2.032	0.003

**Table 2:** Key proteins associated with the underexpressed pathways in hyh mice 14 days after BM-MSC treatment at P4. Sum PEP score corresponds to the score calculated based on the posterior error probability (PEP) values of the peptide spectrum matches (PSM). Sum PEP score indicates the probability that an observed PSM is incorrect. Abundance ratio (AR) indicates the relation of the protein expression in BM-MSC-transplanted mice/sham-injected hyh mice.

Protein	Sum PEP Score	AR	AR *p* Value
ATP-sensitive inward rectifier potassium channel 10	19.789	0.563	0.0008
cAMP-specific 3′.5′-cyclic phosphodiesterase 4A	41.45	0.576	0.014
Cathepsin Z	13.292	0.382	0.031
Complement C1q subcomponent subunit A	22.698	0.466	0.002
Complement C1q subcomponent subunit B	30.001	0.701	0.014
Complement C1q subcomponent subunit C	30.796	0.419	0.002
Fibrinogen beta chain	190.931	0.498	0.02
Fibrinogen gamma chain	108.708	0.442	0.013
Heat shock protein beta-1	31.513	0.383	0.01
Inter-alpha-trypsin inhibitor heavy chain H3	17.338	0.444	0.008
Probable ergosterol biosynthetic protein 28	4.042	0.205	0.024
Protein S100-A11	8.482	0.507	0.049
Serine protease inhibitor A3K	117.573	0.332	0.004
Switch-associated protein 70	7.563	0.464	0.009

**Table 3:** Primary antibodies used for immunolabeling. Chromotek; Planegg-Martinsried, Germany. DSHB; Developmental Studies Hybridoma Bank, Iowa City, IA, USA. Promega; Madison, WI, USA. Abbreviations: I, immunofluorescence or immunohistochemistry; WB, Western blot.

Antibody	Source, Reference	Type	Dilution, Use
AQP4	Sigma-Aldrich, A5971	Rabbit polyclonal	1:200, I, WB
AQP4	Sigma-Aldrich AMAB90537	Mouse monoclonal	1:100, I
BDNF	Abcam, ab108319	Rabbit monoclonal	1:250, I
BrdU	DHSB, G3G4	Mouse monoclonal	1:1000, I
GAPDH	Abcam, ab9485	Rabbit polyclonal	1:1000, WB
GAPDH	Cell Signaling, 5174S	Rabbit monoclonal	1:500, WB
GDNF	Santa Cruz, sc-328	Rabbit polyclonal	1:100, I
GFAP	Sigma-Aldrich, G-A-5	Mouse monoclonal	1:1000, I
GFAP	Sigma-Aldrich, C9205	Mouse monoclonal (Cy3 conjugate)	1:1000, WB
GFAP	Abcam, ab7260	Rabbit polyclonal	1:400, I in vitro
δ-GFAP	Merck Millipore, AB9598	Rabbit polyclonal	1:500, I
Kidins220	Teresa Iglesias [36]	Rabbit polyclonal (TIV24 batch)	1:500, I
NG2	Abcam, ab5320	Rabbit polyclonal	1:200, I
NGF	Abcam, ab6199	Rabbit polyclonal	1:500, I
Rat-401 (nestin)	DHSB, AB 2235915	Mouse monoclonal	1:100, I
mRFP1	Chromotek, 5F8	Rat monoclonal	1:500, I
β–III tubulin	Promega, A6712	Mouse monoclonal	1:5000, I
VEGF	Abcam, ab46154	Rabbit polyclonal	1:1000, I

## Data Availability

The datasets used and/or analyzed during the current study are available from the corresponding author on reasonable request.

## Supplementary Materials

The following supporting information can be downloaded at: https://www.mdpi.com/article/10.3390/ijms24065640/s1.

## References

[1] Kahle K.T., Kulkarni A.V., Limbrick D.D., Warf B.C. (2016). Hydrocephalus in Children. Lancet.

[2] Furey C.G., Antwi P., Kahle K.T., Limbrick D.D., Leonard J.R. (2019). Congenital Hydrocephalus. Cerebrospinal Fluid Disorders.

[3] Garcia-Bonilla M., McAllister J., Limbrick D. (2021). Genetics and Molecular Pathogenesis of Human Hydrocephalus. Neurol. India.

[4] Shirane R., Sato S., Sato K., Kameyama M., Ogawa A., Yoshimoto T., Hatazawa J., Ito M. (1992). Cerebral Blood Flow and Oxygen Metabolism in Infants with Hydrocephalus. Childs Nerv. Syst..

[5] Da Silva M.C., Cinalli G., Sainte-Rose C., Maixner W.J. (2005). Pathophysiology of Hydrocephalus. Pediatric Hydrocephalus.

[6] McAllister J.P. (2012). Pathophysiology of Congenital and Neonatal Hydrocephalus. Semin. Fetal Neonatal Med..

[7] Sansone J.M., Iskandar B.J. (2005). Endoscopic Cerebral Aqueductoplasty: A Trans-Fourth Ventricle Approach. J. Neurosurg..

[8] McAllister J.P., Williams M.A., Walker M.L., Kestle J.R.W., Relkin N.R., Anderson A.M., Gross P.H., Browd S.R. (2015). Hydrocephalus Symposium Expert Panel An Update on Research Priorities in Hydrocephalus: Overview of the Third National Institutes of Health-Sponsored Symposium “Opportunities for Hydrocephalus Research: Pathways to Better Outcomes”. J. Neurosurg..

[9] Tourdias T., Dragonu I., Fushimi Y., Deloire M.S.A., Boiziau C., Brochet B., Moonen C., Petry K.G., Dousset V. (2009). Aquaporin 4 Correlates with Apparent Diffusion Coefficient and Hydrocephalus Severity in the Rat Brain: A Combined MRI-Histological Study. Neuroimage.

[10] Skjolding A.D., Holst A.V., Broholm H., Laursen H., Juhler M. (2013). Differences in Distribution and Regulation of Astrocytic Aquaporin-4 in Human and Rat Hydrocephalic Brain. Neuropathol. Appl. Neurobiol..

[11] Skjolding A.D., Rowland I.J., Søgaard L.V., Praetorius J., Penkowa M., Juhler M. (2010). Hydrocephalus Induces Dynamic Spatiotemporal Regulation of Aquaporin-4 Expression in the Rat Brain. Cereb. Fluid Res..

[12] Owler B.K., Pitham T., Wang D. (2010). Aquaporins: Relevance to Cerebrospinal Fluid Physiology and Therapeutic Potential in Hydrocephalus. Cereb. Fluid Res..

[13] Verkman A.S., Tradtrantip L., Smith A.J., Yao X. (2017). Aquaporin Water Channels and Hydrocephalus. Pediatr. Neurosurg..

[14] Desai B., Hsu Y., Schneller B., Hobbs J.G., Mehta A.I., Linninger A. (2016). Hydrocephalus: The Role of Cerebral Aquaporin-4 Channels and Computational Modeling Considerations of Cerebrospinal Fluid. Focus.

[15] Henzi R., Vío K., Jara C., Johanson C.E., McAllister J.P., Rodríguez E.M., Guerra M. (2020). Neural Stem Cell Therapy of Foetal Onset Hydrocephalus Using the HTx Rat as Experimental Model. Cell Tissue Res..

[16] Ahn S.Y., Chang Y.S., Sung D.K., Sung S.I., Yoo H.S., Lee J.H., Oh W.I., Park W.S. (2013). Mesenchymal Stem Cells Prevent Hydrocephalus after Severe Intraventricular Hemorrhage. Stroke.

[17] García-Bonilla M., Ojeda-Pérez B., García-Martín M.L., Muñoz-Hernández M.C., Vitorica J., Jiménez S., Cifuentes M., Santos-Ruíz L., Shumilov K., Claros S. (2020). Neocortical Tissue Recovery in Severe Congenital Obstructive Hydrocephalus after Intraventricular Administration of Bone Marrow-Derived Mesenchymal Stem Cells. Stem Cell Res. Ther..

[18] Volarevic V., Gazdic M., Simovic Markovic B., Jovicic N., Djonov V., Arsenijevic N. (2017). Mesenchymal Stem Cell-Derived Factors: Immuno-Modulatory Effects and Therapeutic Potential. Biofactors.

[19] Sordi V., Malosio M.L., Marchesi F., Mercalli A., Melzi R., Giordano T., Belmonte N., Ferrari G., Leone B.E., Bertuzzi F. (2005). Bone Marrow Mesenchymal Stem Cells Express a Restricted Set of Functionally Active Chemokine Receptors Capable of Promoting Migration to Pancreatic Islets. Blood.

[20] Volkman R., Offen D. (2017). Concise Review: Mesenchymal Stem Cells in Neurodegenerative Diseases. Stem Cells.

[21] Ghannam S., Bouffi C., Djouad F., Jorgensen C., Noël D. (2010). Immunosuppression by Mesenchymal Stem Cells: Mechanisms and Clinical Applications. Stem Cell Res. Ther..

[22] Bronson R.T., Lane P.W. (1990). Hydrocephalus with Hop Gait (Hyh): A New Mutation on Chromosome 7 in the Mouse. Brain Res. Dev. Brain Res..

[23] Jiménez A.J., Tomé M., Páez P., Wagner C., Rodríguez S., Fernández-Llebrez P., Rodríguez E.M., Pérez-Fígares J.M. (2001). A Programmed Ependymal Denudation Precedes Congenital Hydrocephalus in the Hyh Mutant Mouse. J. Neuropathol. Exp. Neurol..

[24] Wagner C., Bátiz L.F., Rodríguez S., Jiménez A.J., Páez P., Tomé M., Pérez-Fígares J.M., Rodríguez E.M. (2003). Cellular Mechanisms Involved in the Stenosis and Obliteration of the Cerebral Aqueduct of Hyh Mutant Mice Developing Congenital Hydrocephalus. J. Neuropathol. Exp. Neurol..

[25] Páez P., Bátiz L.F., Roales-Buján R., Rodríguez-Pérez L.M., Rodríguez S., Jiménez A.J., Rodríguez E.M., Pérez-Fígares J.M. (2007). Patterned Neuropathologic Events Occurring in Hyh Congenital Hydrocephalic Mutant Mice. J. Neuropathol. Exp. Neurol..

[26] García-Bonilla M., García-Martín M.L., Muñoz-Hernández M.C., Domínguez-Pinos D., Martínez-León M.I., Peñalver A., Castilla L., Alonso F.J., Márquez J., Shumilov K. (2018). A Distinct Metabolite Profile Correlates with Neurodegenerative Conditions and the Severity of Congenital Hydrocephalus. J. Neuropathol. Exp. Neurol..

[27] Roales-Bujan R., Páez P., Guerra M., Rodríguez S., Vío K., Ho-Plagaro A., García-Bonilla M., Rodríguez-Pérez L.M., Domínguez-Pinos M.D., Rodríguez E.M. (2012). Astrocytes Acquire Morphological and Functional Characteristics of Ependymal Cells Following Disruption of Ependyma in Hydrocephalus. Acta Neuropathol..

[28] Domínguez-Pinos M.D., Páez P., Jiménez A.J., Weil B., Arráez M.A., Pérez-Fígares J.M., Rodríguez E.M. (2005). Ependymal Denudation and Alterations of the Subventricular Zone Occur in Human Fetuses with a Moderate Communicating Hydrocephalus. J. Neuropathol. Exp. Neurol..

[29] McAllister J.P., Guerra M.M., Ruiz L.C., Jimenez A.J., Dominguez-Pinos D., Sival D., den Dunnen W., Morales D.M., Schmidt R.E., Rodriguez E.M. (2017). Ventricular Zone Disruption in Human Neonates With Intraventricular Hemorrhage. J. Neuropathol. Exp. Neurol..

[30] Jiménez A.J., Domínguez-Pinos M.D., Guerra M.M., Fernández-Llebrez P., Pérez-Fígares J.M. (2014). Structure and Function of the Ependymal Barrier and Diseases Associated with Ependyma Disruption. Tissue Barriers.

[31] Bátiz L.F., Páez P., Jiménez A.J., Rodríguez S., Wagner C., Pérez-Fígares J.M., Rodríguez E.M. (2006). Heterogeneous Expression of Hydrocephalic Phenotype in the Hyh Mice Carrying a Point Mutation in Alpha-SNAP. Neurobiol. Dis..

[32] Apte R.S., Chen D.S., Ferrara N. (2019). VEGF in Signaling and Disease: Beyond Discovery and Development. Cell.

[33] Zacharek A., Chen J., Li A., Cui X., Li Y., Roberts C., Feng Y., Gao Q., Chopp M. (2007). Angiopoietin1/TIE2 and VEGF/FLK1 Induced by MSC Treatment Amplifies Angiogenesis and Vascular Stabilization after Stroke. J. Cereb. Blood Flow Metab..

[34] Diniz L.P., Matias I., Siqueira M., Stipursky J., Gomes F.C.A. (2019). Astrocytes and the TGF-Β1 Pathway in the Healthy and Diseased Brain: A Double-Edged Sword. Mol. Neurobiol..

[35] Castaneyra-Ruiz L., McAllister J.P., Morales D.M., Brody S.L., Isaacs A.M., Limbrick D.D. (2020). Preterm Intraventricular Hemorrhage in Vitro: Modeling the Cytopathology of the Ventricular Zone. Fluids Barriers CNS.

[36] Del Puerto A., Pose-Utrilla J., Simón-García A., López-Menéndez C., Jiménez A.J., Porlan E., Pajuelo L.S.M., Cano-García G., Martí-Prado B., Sebastián-Serrano Á. (2021). Kidins220 Deficiency Causes Ventriculomegaly via SNX27-Retromer-Dependent AQP4 Degradation. Mol. Psychiatry.

[37] Del Bigio M.R. (2000). Calcium-Mediated Proteolytic Damage in White Matter of Hydrocephalic Rats?. J. Neuropathol. Exp. Neurol..

[38] Braun K.P., Dijkhuizen R.M., de Graaf R.A., Nicolay K., Vandertop W.P., Gooskens R.H., Tulleken K.A. (1997). Cerebral Ischemia and White Matter Edema in Experimental Hydrocephalus: A Combined in Vivo MRI and MRS Study. Brain Res..

[39] Castejón O.J. (2010). Submicroscopic Pathology of Human and Experimental Hydrocephalic Cerebral Cortex. Folia Neuropathol..

[40] Jiménez A.J., Rodríguez-Pérez L.M., Domínguez-Pinos M.D., Gómez-Roldán M.C., García-Bonilla M., Ho-Plagaro A., Roales-Buján R., Jiménez S., Roquero-Mañueco M.C., Martínez-León M.I. (2014). Increased Levels of Tumour Necrosis Factor Alpha (TNFα) but Not Transforming Growth Factor-Beta 1 (TGFβ1) Are Associated with the Severity of Congenital Hydrocephalus in the Hyh Mouse. Neuropathol. Appl. Neurobiol..

[41] Jiménez A.J., García-Verdugo J.M., González C.A., Bátiz L.F., Rodríguez-Pérez L.M., Páez P., Soriano-Navarro M., Roales-Buján R., Rivera P., Rodríguez S. (2009). Disruption of the Neurogenic Niche in the Subventricular Zone of Postnatal Hydrocephalic Hyh Mice. J. Neuropathol. Exp. Neurol..

[42] Pirzad Jahromi G., Shabanzadeh Pirsaraei A., Sadr S.S., Kaka G., Jafari M., Seidi S., Charish J. (2015). Multipotent Bone Marrow Stromal Cell Therapy Promotes Endogenous Cell Proliferation Following Ischemic Stroke. Clin. Exp. Pharmacol. Physiol..

[43] Chen X., Xu C.-X., Liang H., Xi Z., Pan J., Yang Y., Sun Q., Yang G., Sun Y., Bian L. (2020). Bone Marrow Mesenchymal Stem Cells Transplantation Alleviates Brain Injury after Intracerebral Hemorrhage in Mice through the Hippo Signaling Pathway. Aging.

[44] Shen L.H., Li Y., Chopp M. (2010). Astrocytic Endogenous Glial Cell Derived Neurotrophic Factor Production Is Enhanced by Bone Marrow Stromal Cell Transplantation in the Ischemic Boundary Zone after Stroke in Adult Rats. Glia.

[45] Linnerbauer M., Rothhammer V. (2020). Protective Functions of Reactive Astrocytes Following Central Nervous System Insult. Front. Immunol..

[46] Liddelow S.A., Barres B.A. (2017). Reactive Astrocytes: Production, Function, and Therapeutic Potential. Immunity.

[47] Filippidis A.S., Kalani M.Y.S., Rekate H.L. (2011). Hydrocephalus and Aquaporins: Lessons Learned from the Bench. Child’s Nerv. Syst..

[48] Guo J., Mi X., Zhan R., Li M., Wei L., Sun J. (2018). Aquaporin 4 Silencing Aggravates Hydrocephalus Induced by Injection of Autologous Blood in Rats. Med. Sci. Monit..

[49] Castaneyra-Ruiz L., Morales D.M., McAllister J.P., Brody S.L., Isaacs A.M., Strahle J.M., Dahiya S.M., Limbrick D.D. (2018). Blood Exposure Causes Ventricular Zone Disruption and Glial Activation In Vitro. J. Neuropathol. Exp. Neurol..

[50] Tang G., Yang G.-Y. (2016). Aquaporin-4: A Potential Therapeutic Target for Cerebral Edema. Int. J. Mol. Sci..

[51] Milhorat T.H. (1992). Classification of the Cerebral Edemas with Reference to Hydrocephalus and Pseudotumor Cerebri. Child’s Nerv. Syst..

[52] Iencean S.M. (2003). Brain Edema—A New Classification. Med. Hypotheses.

[53] Das M., Mayilsamy K., Mohapatra S.S., Mohapatra S. (2019). Mesenchymal Stem Cell Therapy for the Treatment of Traumatic Brain Injury: Progress and Prospects. Rev. Neurosci..

[54] Li N., Wang P., Ma X.-L., Wang J., Zhao L.-J., Du L., Wang L.-Y., Wang X.-R., Liu K.-D. (2014). Effect of Bone Marrow Stromal Cell Transplantation on Neurologic Function and Expression of VEGF in Rats with Focal Cerebral Ischemia. Mol. Med. Rep..

[55] Chang Y.S., Ahn S.Y., Jeon H.B., Sung D.K., Kim E.S., Sung S.I., Yoo H.S., Choi S.J., Oh W.I., Park W.S. (2014). Critical Role of Vascular Endothelial Growth Factor Secreted by Mesenchymal Stem Cells in Hyperoxic Lung Injury. Am. J. Respir. Cell Mol. Biol..

[56] Chuang T.J., Lin K.C., Chio C.C., Wang C.C., Chang C.P., Kuo J.R. (2012). Effects of Secretome Obtained from Normoxia-Preconditioned Human Mesenchymal Stem Cells in Traumatic Brain Injury Rats. J. Trauma Acute Care Surg..

[57] Cho S.R., Suh H., Yu J.H., Kim H.H., Seo J.H., Seo C.H. (2016). Astroglial Activation by an Enriched Environment after Transplantation of Mesenchymal Stem Cells Enhances Angiogenesis after Hypoxic-Ischemic Brain Injury. Int. J. Mol. Sci..

[58] Madrigal M., Rao K.S., Riordan N.H. (2014). A Review of Therapeutic Effects of Mesenchymal Stem Cell Secretions and Induction of Secretory Modification by Different Culture Methods. J. Transl. Med..

[59] Pisani F., Cammalleri M., Dal Monte M., Locri F., Mola M.G., Nicchia G.P., Frigeri A., Bagnoli P., Svelto M. (2018). Potential Role of the Methylation of VEGF Gene Promoter in Response to Hypoxia in Oxygen-Induced Retinopathy: Beneficial Effect of the Absence of AQP4. J. Cell. Mol. Med..

[60] Kaur C., Sivakumar V., Yong Z., Lu J., Foulds W.S., Ling E.A. (2007). Blood-Retinal Barrier Disruption and Ultrastructural Changes in the Hypoxic Retina in Adult Rats: The Beneficial Effect of Melatonin Administration. J. Pathol..

[61] Kaur C., Sivakumar V., Zhang Y., Ling E.A. (2006). Hypoxia-Induced Astrocytic Reaction and Increased Vascular Permeability in the Rat Cerebellum. Glia.

[62] Zou Y.Y., Lu J., Poon D.J.F., Kaur C., Cao Q., Teo A.L., Ling E.A. (2009). Combustion Smoke Exposure Induces Up-Regulated Expression of Vascular Endothelial Growth Factor, Aquaporin 4, Nitric Oxide Synthases and Vascular Permeability in the Retina of Adult Rats. Neuroscience.

[63] Do P.T., Wu C.-C., Chiang Y.-H., Hu C.-J., Chen K.-Y. (2021). Mesenchymal Stem/Stromal Cell Therapy in Blood–Brain Barrier Preservation Following Ischemia: Molecular Mechanisms and Prospects. Int. J. Mol. Sci..

[64] Yan H., Chen Y., Li L., Jiang J., Wu G., Zuo Y., Zhang J.H., Feng H., Yan X., Liu F. (2016). Decorin Alleviated Chronic Hydrocephalus via Inhibiting TGF-Β1/Smad/CTGF Pathway after Subarachnoid Hemorrhage in Rats. Brain Res..

[65] Botfield H., Gonzalez A.M., Abdullah O., Skjolding A.D., Berry M., McAllister J.P., Logan A. (2013). Decorin Prevents the Development of Juvenile Communicating Hydrocephalus. Brain.

[66] Kitazawa K., Tada T. (1994). Elevation of Transforming Growth Factor-Beta 1 Level in Cerebrospinal Fluid of Patients with Communicating Hydrocephalus after Subarachnoid Hemorrhage. Stroke.

[67] Cai X., Pattisapu J., Tarnuzzer R., Fernandez-Valle C., Gibson J. (1999). TGF-Β1 Expression Is Reduced in Hydrocephalic H-Tx Rat Brain. Eur. J. Pediatr. Surg..

[68] Cekanaviciute E., Fathali N., Doyle K.P., Williams A.M., Han J., Buckwalter M.S. (2014). Astrocytic Transforming Growth Factor-Beta Signaling Reduces Subacute Neuroinflammation after Stroke in Mice: Astrocytic TGFβ Reduces Neuroinflammation. Glia.

[69] Zhu Y., Yang G.-Y., Ahlemeyer B., Pang L., Che X.-M., Culmsee C., Klumpp S., Krieglstein J. (2002). Transforming Growth Factor-Β1 Increases Bad Phosphorylation and Protects Neurons Against Damage. J. Neurosci..

[70] Yu G., Fahnestock M. (2002). Differential Expression of Nerve Growth Factor Transcripts in Glia and Neurons and Their Regulation by Transforming Growth Factor-Β1. Mol. Brain Res..

[71] Semkova I., Krieglstein J. (1999). Neuroprotection Mediated via Neurotrophic Factors and Induction of Neurotrophic Factors. Brain Res. Rev..

[72] Saher G., Stumpf S.K. (2015). Cholesterol in Myelin Biogenesis and Hypomyelinating Disorders. Biochim. Biophys. Acta.

[73] Bashaw G.J., Klein R. (2010). Signaling from Axon Guidance Receptors. Cold Spring Harb. Perspect. Biol..

[74] Huber A.B., Kolodkin A.L., Ginty D.D., Cloutier J.F. (2003). Signaling at the Growth Cone: Ligand-Receptor Complexes and the Control of Axon Growth and Guidance. Annu. Rev. Neurosci..

[75] Eroglu C., Barres B.A. (2010). Regulation of Synaptic Connectivity by Glia. Nature.

[76] Cao N., Liao T., Liu J., Fan Z., Zeng Q., Zhou J., Pei H., Xi J., He L., Chen L. (2017). Clinical-Grade Human Umbilical Cord-Derived Mesenchymal Stem Cells Reverse Cognitive Aging via Improving Synaptic Plasticity and Endogenous Neurogenesis. Cell Death Dis..

[77] Batiz L.F., Roales-Buján R., Rodríguez-Pérez L.M., Matas I.M., Páez P., Roque M., Jiménez A.J., Ramos C., Pérez-Fígares J.M. (2009). A Simple PCR-Based Genotyping Method for M105I Mutation of Alpha-SNAP Enhances the Study of Early Pathological Changes in Hyh Phenotype. Mol. Cell. Probes.

[78] Schindelin J., Arganda-Carreras I., Frise E., Kaynig V., Longair M., Pietzsch T., Preibisch S., Rueden C., Saalfeld S., Schmid B. (2012). Fiji: An Open-Source Platform for Biological-Image Analysis. Nat. Methods.

[79] Schneider C.A., Rasband W.S., Eliceiri K.W. (2012). NIH Image to ImageJ: 25 Years of Image Analysis. Nat. Methods.

