# Supplementary material for: Generation of Periventricular Reactive Astrocytes Overexpressing Aquaporin 4 Is Stimulated by Mesenchymal Stem Cell Therapy
Source: Int J Mol Sci. 2023 Mar 15;24(6):5640. doi: 10.3390/ijms24065640 (PMC10057840; doi:10.3390/ijms24065640)
Supplement: Supplementary file 1 [file ijms-24-05640-s001.zip › FIGURE S2.pdf]

FIGURE S2

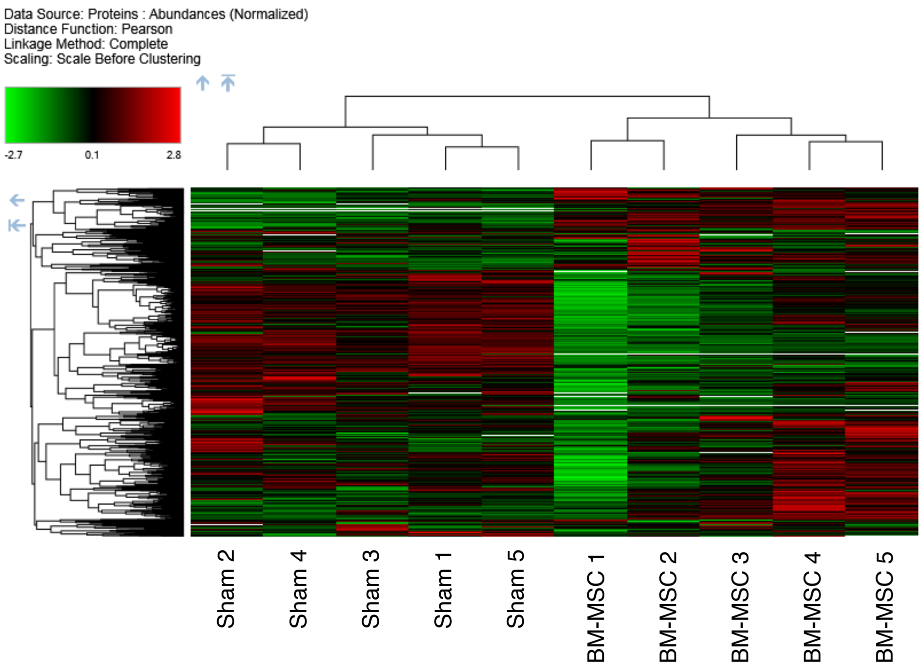

a

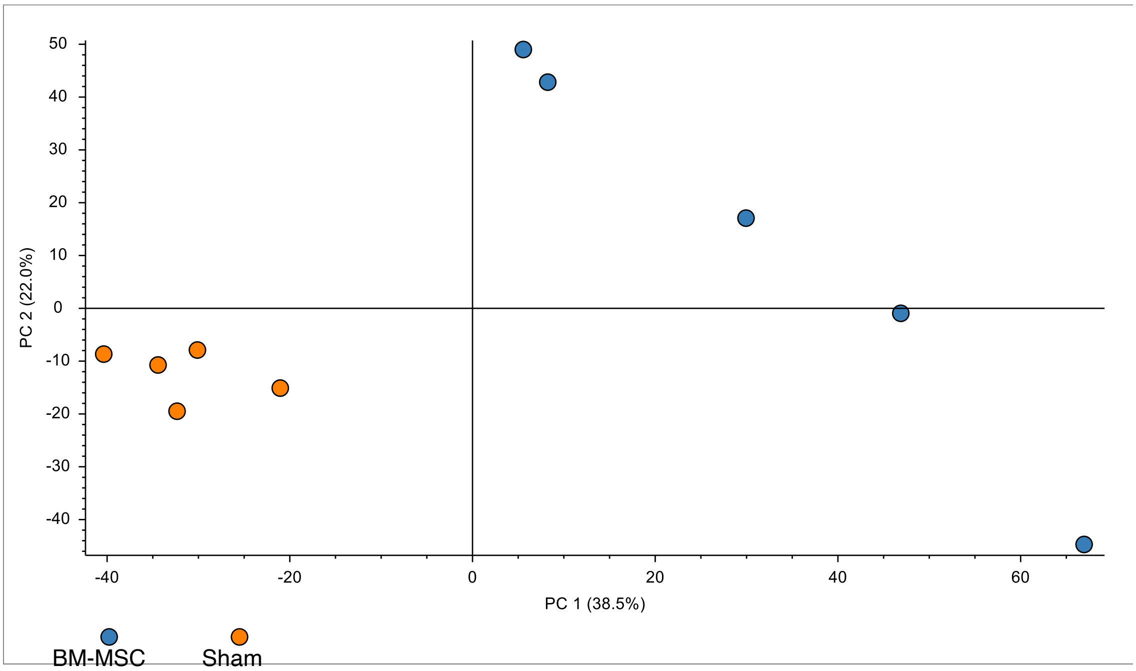

b

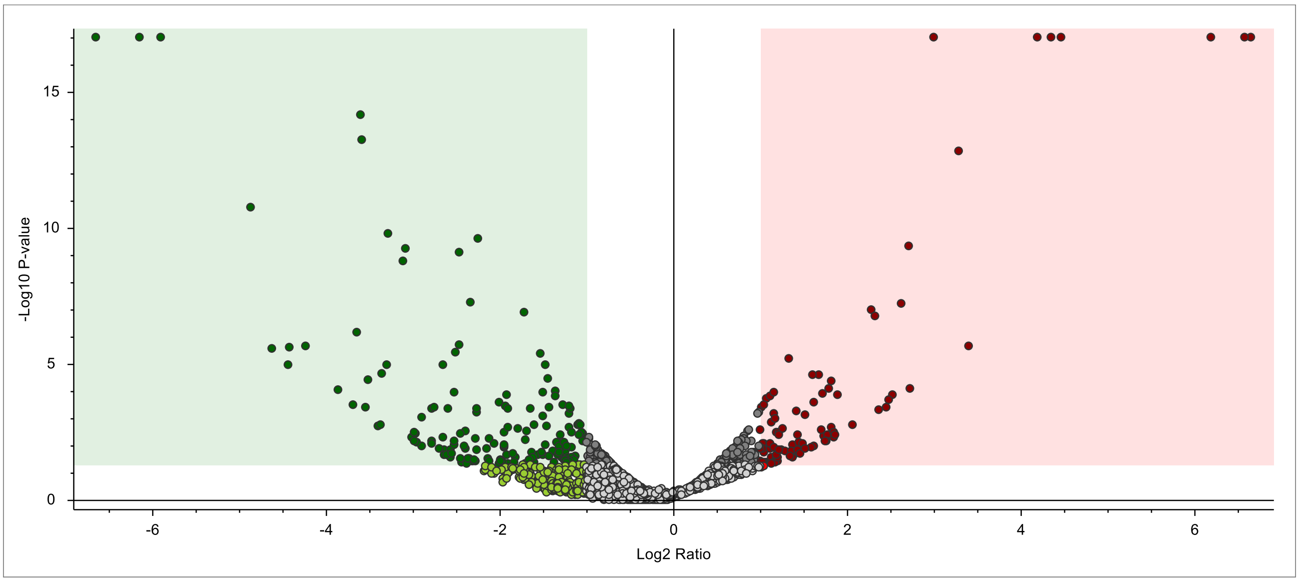

c
