# Supplementary material for: Generation of Periventricular Reactive Astrocytes Overexpressing Aquaporin 4 Is Stimulated by Mesenchymal Stem Cell Therapy
Source: Int J Mol Sci. 2023 Mar 15;24(6):5640. doi: 10.3390/ijms24065640 (PMC10057840; doi:10.3390/ijms24065640)
Supplement: Supplementary file 1 [file ijms-24-05640-s001.zip › FIGURE S3.pdf]

FIGURE S3

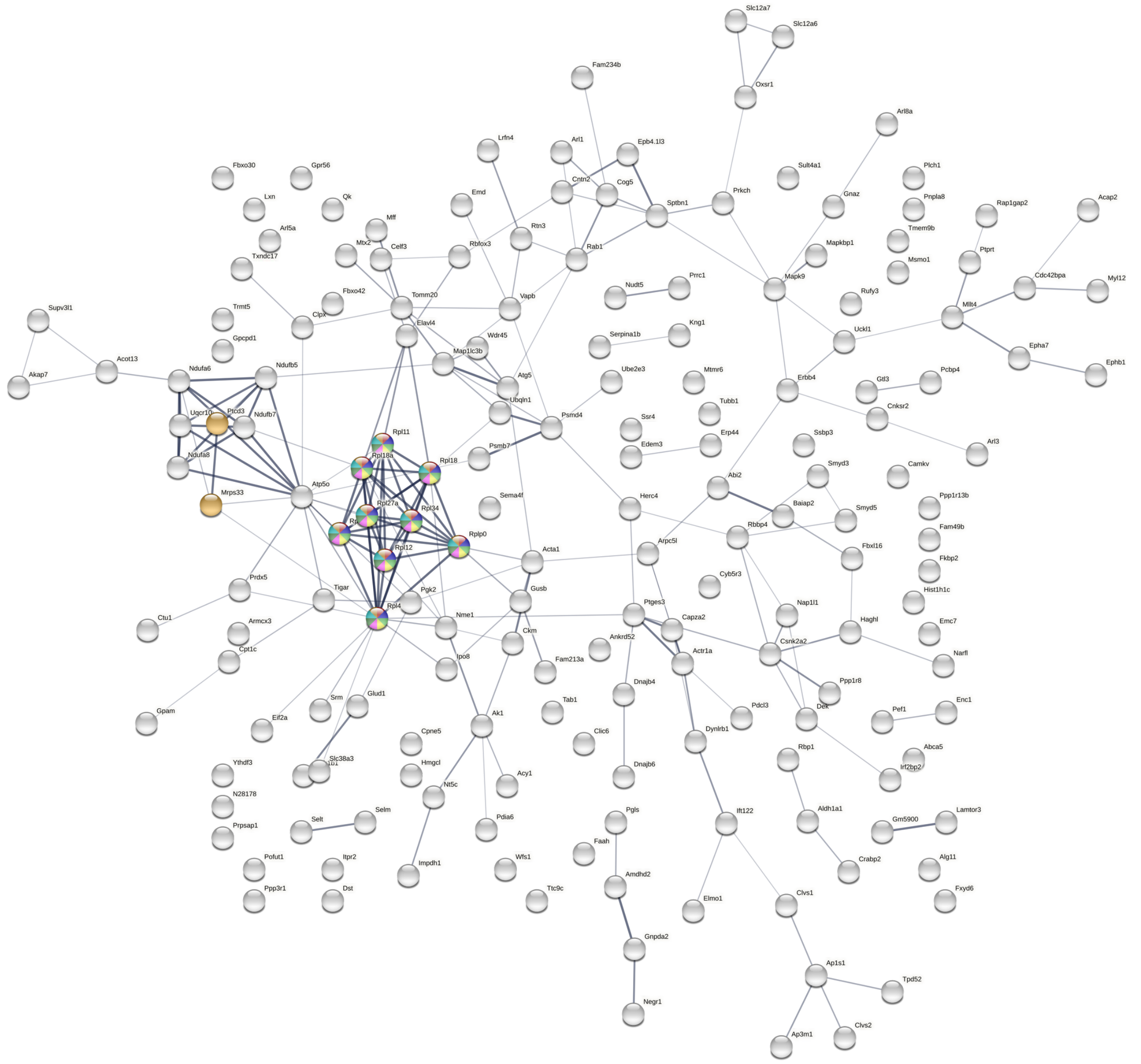

MMU-1799339 SRP-dependent cotranslational protein targeting to membrane
Strength: 1.09; False Discovery rate: 0.00095

MMU-975956 Nonsense Mediated Decay (NMD) independent of the Exon Junction Complex (EJC)
Strength: 1.08 ; False Discovery rate: 0.00095

MMU-72689 Formation of a pool of free 40S subunits
Strength: 1.05; False Discovery rate: 0.0010

MMU-156827 L13a-mediated translational silencing of Ceruloplasmin expression
Strength: 1.01; False Discovery rate: 0.0018

MMU-72706 GTP hydrolysis and joining of the 60S ribosomal subunit
Strength: 1.0; False Discovery rate: 0.0018

MMU-975957 Nonsense Mediated Decay (NMD) enhanced by the Exon Junction Complex (EJC)
Strength: 0.99; False Discovery rate: 0.0018

MMU-6791226 Major pathway of rRNA processing in the nucleolus and cytosol
Strength: 0.8; False Discovery rate: 0.0254

MMU-72766 Translation
Strength: 0.77; False Discovery rate: 0.0064

Strength:
Log10(observed / expected). This measure describes how large the enrichment effect is. It's the ratio between i) the number of proteins in your network that are annotated with a term and ii) the number of proteins that we expect to be annotated with this term in a random network of the same size.

False Discovery Rate:
This measure describes how significant the enrichment is. Shown are p-values corrected for multiple testing within each category using the Benjamini–Hochberg procedure.
