# Supplementary material for: Generation of Periventricular Reactive Astrocytes Overexpressing Aquaporin 4 Is Stimulated by Mesenchymal Stem Cell Therapy
Source: Int J Mol Sci. 2023 Mar 15;24(6):5640. doi: 10.3390/ijms24065640 (PMC10057840; doi:10.3390/ijms24065640)
Supplement: Supplementary file 1 [file ijms-24-05640-s001.zip › ijms-2236263-supplementary.pdf]

Figure S1. (**a**) Percentage of area represented by blood vessels in hematoxylin-eosin paraffin-stained sections (representative images shown in **b** and **c**) from sham-injected hyh mice (*sham*, n = 13) and BM-MSC-transplanted (*BM-MSC*, n = 17) hydrocephalic hyh mice at P18.

Figure S2. Differential protein expression analysis in sham-injected hydrocephalic hyh mice (sham, n = 5) and BM-MSCs-transplanted (BM-MSC, n = 5) hydrocephalic hyh mice at P8. (**a**) Heatmaps and clusters of the two groups of mice. (**b**) Principal component analysis of hyh mice treated with BM-MSCs and hyh mice. (**c**) Volcano representation for overexpressed and underexpressed proteins in BM-MSC-treated mice compared with sham-injected hyh mice.

Figure S3. Overexpressed proteins analyzed and represented with STRING comparing BM-MSC-transplanted (BM-MSC, n = 5) hydrocephalic hyh mice with sham-injected hydrocephalic hyh mice (sham, n = 5) at P8. The results of Reactome pathways (MMU) are shown. *Strength* represents log10 (observed/expected). This measure describes how large the enrichment effect is. It is the ratio between i) the number of proteins in your network that are annotated with a term and ii) the number of proteins that we expect to be annotated with this term in a random network of the same size. *False discovery rate* describes how significant the enrichment is. The p-values corrected for multiple testing within each category using the Benjamini–Hochberg procedure are shown.
