# Supplementary figures and images for: Generation of Periventricular Reactive Astrocytes Overexpressing Aquaporin 4 Is Stimulated by Mesenchymal Stem Cell Therapy
Source: Int J Mol Sci. 2023 Mar 15;24(6):5640. doi: 10.3390/ijms24065640 (PMC10057840; doi:10.3390/ijms24065640)

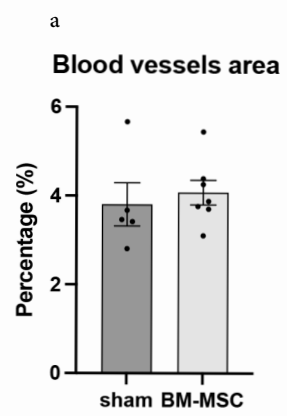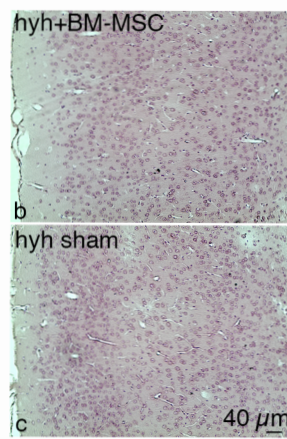

Supplement: Supplementary file 1 [file ijms-24-05640-s001.zip › FIGURE S1.pdf]
